# A therapeutic role for a regulatory GLUT1–associated lncRNA in GLUT1-deficient mice

**DOI:** 10.1172/JCI193519

**Published:** 2026-03-05

**Authors:** Maoxue Tang, Sasa Teng, Yueqing Peng, Ashley Y. Kim, Yoon-Ra Her, Peter Canoll, Jeffrey N. Bruce, Phyllis L. Faust, Kailash Adhikari, Darryl C. De Vivo, Umrao R. Monani

**Affiliations:** 1 Department of Neurology,; 2 Center for Motor Neuron Biology and Disease,; 3 Colleen Giblin Research Laboratories,; 4 Department of Pathology & Cell Biology, and; 5 Department of Neurological Surgery, Columbia University Irving Medical Center, New York, New York, USA.; 6 Sarepta Therapeutics Inc. Cambridge, Masschusetts, USA.

**Keywords:** Genetics, Neuroscience, Monogenic diseases, Mouse models, Neurological disorders

## Abstract

The mammalian brain relies primarily on glucose for its energy needs. Delivery of this nutrient to the brain is mediated by the glucose transporter-1 (GLUT1) protein. Low GLUT1 thwarts glucose entry into the brain, causing an energy crisis and triggering, in one instance, the debilitating neurodevelopmental condition known as GLUT1 deficiency syndrome (GLUT1DS). Current treatments for GLUT1DS are suboptimal, as none address the root cause — low GLUT1 — of the condition. Levels of this transporter must respond rapidly to the brain’s changing energy requirements. This necessitates fine tuning its expression. Here, we describe a long-noncoding RNA (lncRNA) antisense to *GLUT1 (SLC2A1)* and show that it is involved in such regulation. Raising levels of the lncRNA had a concordant effect on GLUT1 in cultured human cells and transgenic mice; reducing levels elicited the opposite effect. Delivering the lncRNA to GLUT1DS model mice via viral vectors induced GLUT1 expression, enhancing brain glucose levels to mitigate disease. Direct delivery of such a lncRNA to combat disease has not been reported previously and constitutes, to our knowledge, a unique therapeutic paradigm. Moreover, considering the importance of maintaining homeostatic GLUT1 levels, calibrating transporter expression via the lncRNA could become broadly relevant to myriad conditions, including Alzheimer’s disease, wherein GLUT1 is perturbed.

## Abstract 2

Here we report a regulatory molecule that modulates nutrient delivery to the brain and show that it has therapeutic value for brain energy failure syndromes.

## Graphical abstract

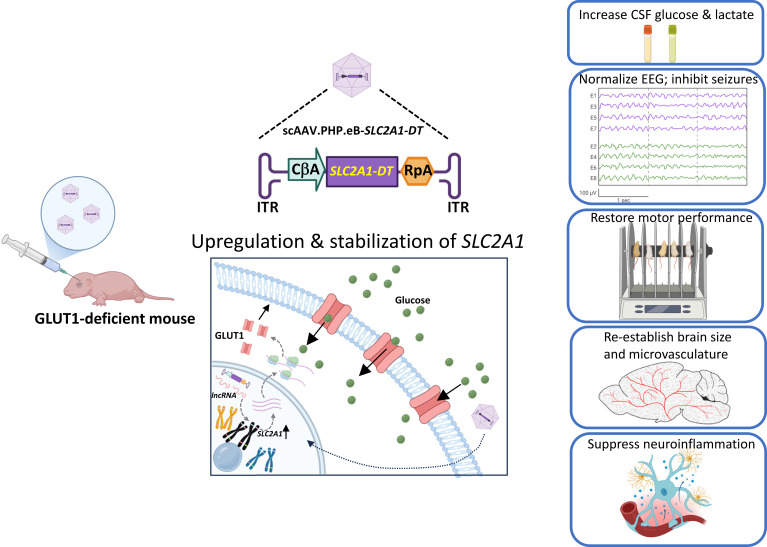

## Introduction

Although it accounts for just 2% of the weight of the average adult, the human brain has an unusually high energy requirement, consuming approximately 25% of all nutrient intake (1). This energy is supplied to the brain mainly in the form of glucose and delivered to the cerebral parenchyma via the glucose transporter1 (GLUT1) protein (2). Complete loss of GLUT1 causes death in utero in mice (3) and is almost certainly embryonic lethal in humans, too. In contrast, low (approximately 50%) GLUT1, while sufficient to ensure embryonic development, causes an energy crisis — neuroglycopenia — which is particularly detrimental to the brain and CNS (4, 5). Understanding and combating neuroglycopenia is broadly relevant, as it characterizes several common conditions, including Alzheimer’s disease (AD) and traumatic brain encephalopathies (TBEs) (6). Yet, it is perhaps best investigated in and exemplified by the neurodevelopmental disorder GLUT1 deficiency syndrome (GLUT1DS) (7). Resulting from *SLC2A1* *(GLUT1)* haploinsufficiency — mostly a consequence of de novo mutations — GLUT1DS is reported to occur as frequently as 1 in 24,000 newborns (8–10). Individuals afflicted with the condition classically become symptomatic during infancy, exhibiting a complex phenotype that initially manifests as abnormal eye-head movements, intractable epileptic seizures, and neurodevelopmental delay (11). A complex movement disorder that combines elements of ataxia, dystonia, and spasticity often develops later in life and can be complicated by exercise-induced dyskinesia and hemolytic anemia (7).

Although the genetic defect underlying GLUT1DS was identified more than 2 decades ago, there is still no treatment that directly addresses GLUT1 deficiency. Ketogenic diets, which supply the brain with an alternate, albeit imperfect, energy source, ketone bodies, are the current mainstay for individuals diagnosed with the condition (7). Such diets mitigate seizure activity in patients but often fail to prevent movement disorders and, in some instances, trigger serious adverse effects, e.g., marked reduction of bone mass, long-term cardiovascular complications due to atherosclerosis and, in rare instances, the induction of a state of coma (12–14). GLUT1DS thus remains a disease with an urgent unmet medical need.

Considering the link between *SLC2A1* haploinsufficiency and GLUT1DS, one intuitively attractive alternative therapeutic strategy to ketogenic diets that directly addresses the molecular defect in the condition is simply to restore cerebral levels of the transporter. This may be accomplished with small molecules that relieve transcriptional or translational repression but may equally well be brought about by regulatory RNAs. This study focuses on the latter approach and stems from the discovery of an RNA located in the *SLC2A1* locus. The RNA, a natural antisense long noncoding transcript (lncRNA) has a potent effect on GLUT1 expression. Using a combination of cultured human cells, human tissues, transgenic mice, and a well-established rodent model of GLUT1DS, we show that the lncRNA concordantly regulates *SLC2A1,* increasing GLUT1 to therapeutic levels. Indeed, delivering the transcript in a viral vector to model mice raised cerebral GLUT1 sufficiently to prevent the onset of motor defects, ameliorate abnormal brain activity, and suppress the neuroinflammation commonly seen in mutants. This therapeutic modality wherein a natural antisense transcript is employed to stimulate expression of its protein-coding cognate to treat disease is, to our knowledge, novel. Moreover, considering the myriad conditions involving reduced GLUT1, the therapeutic strategy described here for GLUT1DS could become relevant beyond just this rare neurodevelopmental disorder.

## Results

### A natural antisense transcript concordantly regulates human SLC2A1 — GLUT1 — expression.

**Figure 1:**
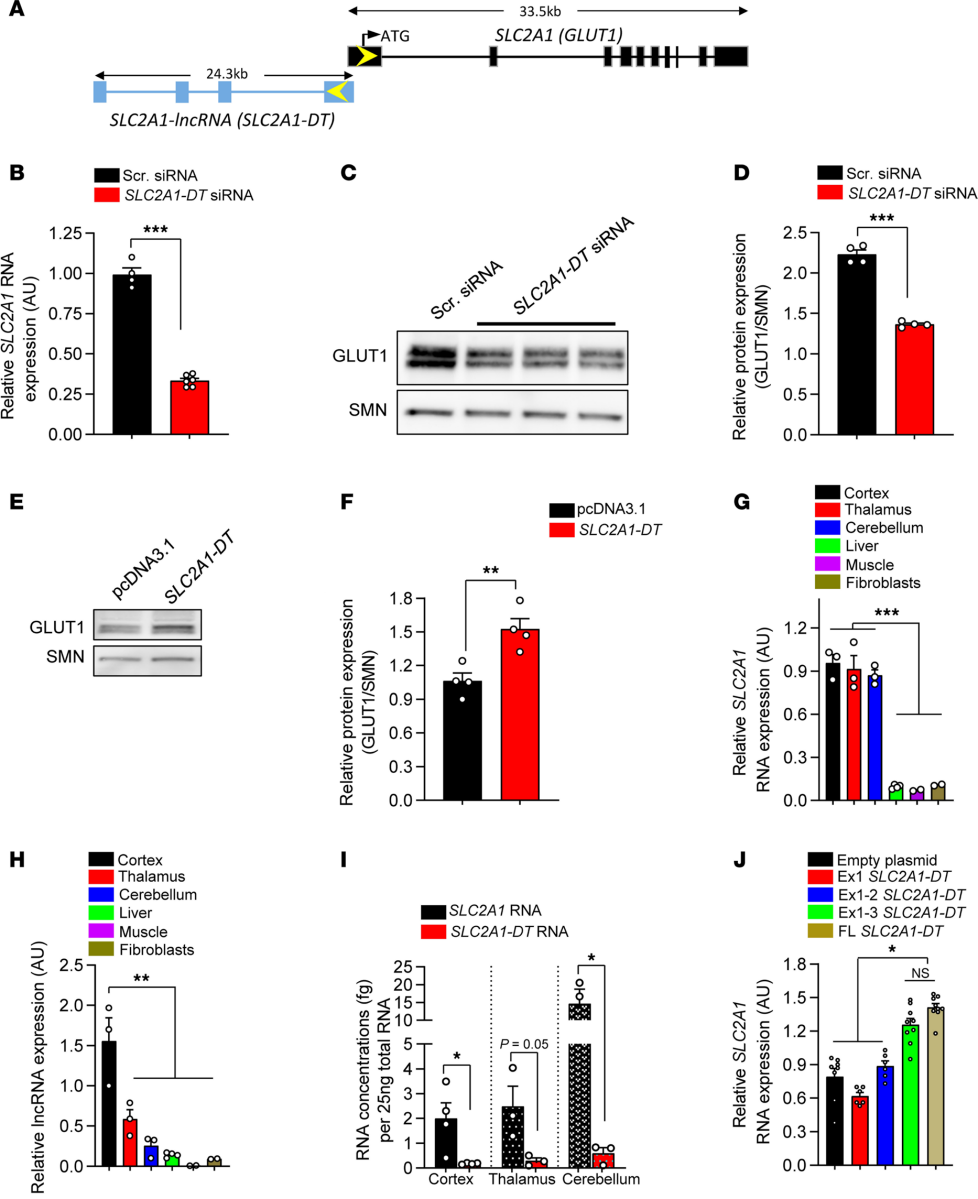
A natural antisense transcript regulates *SLC2A1/GLUT1* expression concordantly. (**A**) Schematic depicting spatial relationship between the *SLC2A1* gene and the *SLC2A1-DT* natural antisense transcript. Exons 1 of *SLC2A1* and *SLC2A1-DT* overlap over 128 bp. (**B**) Quantified results of *SLC2A1-DT* knockdown on *SLC2A1* RNA levels in human fibroblasts. ***P < 0.001, *t* test, *n* ≥ 4 replicates for each condition. (**C**) Representative Western blot showing effect of *SLC2A1-DT* knockdown on GLUT1 protein levels in fibroblasts. (**D**) Quantified results of Western blots to determine effect of suppressing *SLC2A1-DT* expression on GLUT1 protein levels. ***P < 0.001, *t* test, *n* = 4 replicates for each condition. (**E**) Representative Western blot depicting increase in GLUT1 protein in human fibroblasts following overexpression of the *SLC2A1-DT* lncRNA. (**F**) Graph depicting quantified increase of GLUT1 protein when the lncRNA is overexpressed. **P < 0.01, *t* test, *n* = 4 replicates each. Expression profiles of (**G**) *SLC2A1* and (**H**) lncRNA in various human tissues. **P < 0.01, ***P < 0.001, 1-way ANOVA. (**I**) Graph depicting low levels of the lncRNA relative to *SLC2A1* levels in human brain tissues sampled. *P < 0.05, *t* tests, *n* ≥ 3 samples. (**J**) Quantified relative *SLC2A1* RNA levels obtained from experiments to determine the minimum *SLC2A1-DT* exons to promote *SLC2A1* activity. *P < 0.05, Kruskal-Wallis test, *n* ≥ 6 test replicates with each construct.

While examining the human *SLC2A1* locus, we discovered an expressed sequence located 5’ to the *SLC2A1* gene. On closer inspection, the expressed sequence turned out to be a spliced, polyadenylated 1.1 kb natural antisense lncRNA transcript consisting of 4 exons spread over approximately 24 kb of genomic sequence on human Chr.1 (Figure 1A). Considering a growing recognition that such lncRNAs regulate expression of their cognate protein-coding genes (15), we inquired if the transcript — now designated *SLC2A1-DT* in public databases — modulated *SLC2A1* activity. To do so, we first knocked down lncRNA expression using siRNAs (Supplemental Figure 1A; supplemental material available online with this article; https://doi.org/10.1172/JCI193519DS1). One of these (siRNA#2) was subsequently employed to examine the effect of lncRNA knockdown on GLUT1 levels (Figure 1B). Natural antisense transcript (NAT) expression often exhibits a discordant relationship with that of its sense strand partner (16). We were therefore surprised to find that knockdown of the lncRNA produced a concordant effect, resulting in significantly lower *SLC2A1* RNA levels (Figure 1B). Consistent with reduced *SLC2A1* RNA levels, GLUT1 protein also decreased (Figure 1, C and D); predictably, we failed to detect any difference in protein levels between vehicle-treated cells and cells transfected with the scrambled siRNA used as a control (Supplemental Figure 1, B and C). Conversely, overexpressing lncRNA from a plasmid in human fibroblasts raised GLUT1 RNA and protein (Figure 1, E and F), whereas no difference in protein amounts between vehicle-treated cells and cells treated with empty plasmid was detected (Supplemental Figure 1, D and E). The increase in GLUT1 protein when the lncRNA was overexpressed from a plasmid suggested that the element expressing the NAT need not be positioned in cis to bring about an effect. These effects of the *SLC2A1-DT* lncRNA on *SLC2A1* were also observed in human brain endothelial cells (BECs), which express abundant transporter and are important in delivering glucose to the brain parenchyma. Indeed, knocking down and raising lncRNA expression, respectively, inhibited and enhanced *SLC2A1* transcript levels significantly (Supplemental Figure 1, F–H). Finally, consistent with the ability to function in trans, cotransfecting the lncRNA into cultured cells with a construct containing a 6 kb *SLC2A1* promoter fragment driving luciferase stimulated expression of the luciferase reporter (Supplemental Figure 1, I and J). This last result hints at a transcription-enhancing role of the lncRNA.

NATs are typically expressed at low levels, with a tissue specificity reflective of that of their sense strand partners (16, 17). Accordingly, to further characterize *SLC2A1-DT*, we examined its expression and that of *SLC2A1* in diverse human tissue samples. Akin to high GLUT1 levels in brain samples, the lncRNA was expressed especially robustly in the cerebral cortex (Figure 1, G and H). This pattern of concordant expression was also observed in 2 human brain tumor samples wherein characteristically and abnormally high *SLC2A1* transcripts were accompanied by enhanced lncRNA expression, relative to levels in healthy brain tissue (Supplemental Figure 1, K and L). Still, consistent with generally low expression of lncRNAs, *SLC2A1-DT* was found at markedly lower concentrations relative to GLUT1 in the human brain regions that we examined (Figure 1I). In a final set of studies using cultured cells, we inquired if truncated versions of the lncRNA retained the ability to regulate *SLC2A1*. In parallel, we investigated the cellular localization of the transcript. We found that exons 1–3 of the lncRNA were just as effective in raising GLUT1 as the full-length transcript was, whereas constructs containing just exon 1 or the first 2 exons failed to stimulate *SLC2A1* expression (Figure 1J). Experiments to determine the cellular distribution of the lncRNA demonstrated that it localizes to the cytoplasm as well as the nucleus (Supplemental Figure 2). Collectively, these results suggest that *SLC2A1-DT*, a recently annotated NAT in the human *SLC2A1* locus, not only regulates *SLC2A1* expression in a concordant manner but can also do so in trans.

### Transgenic expression of SLC2A1-DT mitigates disease in GLUT1DS-model mice.

**Figure 2:**
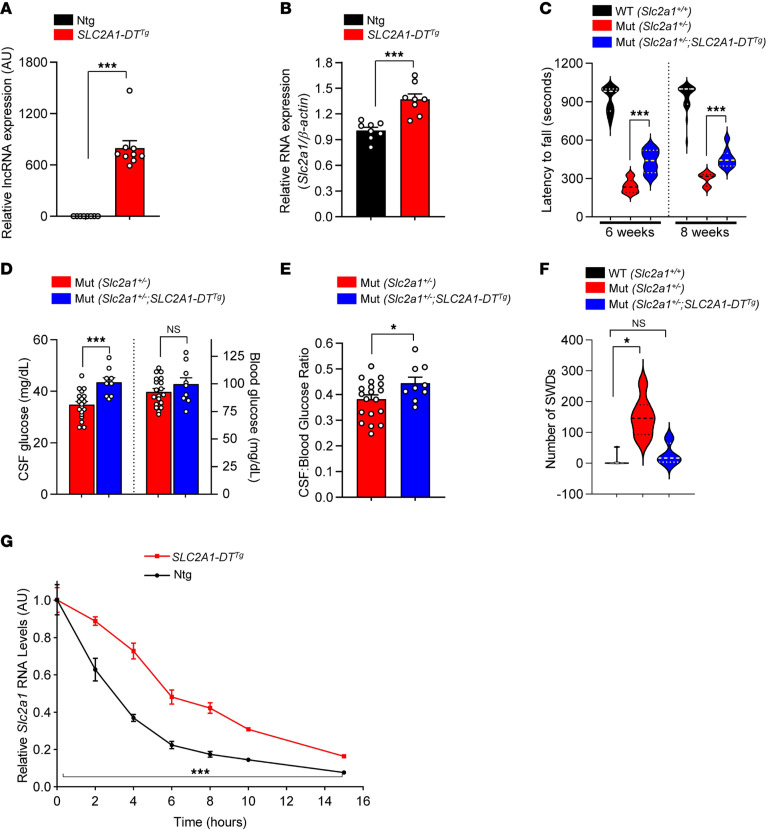
Transgenic expression of the *SLC2A1-DT* lncRNA stabilizes *Slc2a1* transcript and suppresses GLUT1DS in model mice. Quantified expression of (**A**) the lncRNA and (**B**) murine *Slc2a1* in transgenic mice and nontransgenic littermates. ***P < 0.001, *t* test, *n* ≥ 8 mice of each cohort. (**C**) Graph depicting significantly improved motor performance of GLUT1DS mutants harboring the lncRNA transgene. ***P < 0.001, *t* test, *n* = 8 and *n* = 10 mutants, respectively with and without the *SLC2A1-DT^Tg^* transgene; *n* = 35 WT controls. Quantified results of (**D**) CSF and blood glucose level measurements and (**E**) CSF-to-blood glucose ratios in mutants with or without the lncRNA transgene. *P < 0.05, ***P < 0.001, *t* tests, *n* = 9 and 19 mutants with and without *SLC2A1-DT^Tg^*, respectively. (**F**) Graph depicts fewer seizures manifesting as spike-wave discharges in GLUT1DS mutants bearing the lncRNA transgene relative to mutants devoid of the transgene. *P < 0.05, Mann-Whitney test, *n* = 4 *SLC2A1-DT^Tg^* mutants and *n* = 7 mice of each of the other 2 cohorts. (**G**) Graph of murine *Slc2a1* RNA decay in MEFs either WT (Ntg) or transgenic for the *SLC2A1-DT* lncRNA, following treatment with Actinomycin D to halt transcription. ***P < 0.001, AOCs compared using *t* test, results compiled from 3 independent experiments.

Considering the stimulatory effect of the *SLC2A1*-*DT* NAT on *SLC2A1* expression and our long-term objective of developing therapies for GLUT1DS that restore transporter levels to patients afflicted with the condition, we inquired if the NAT also induced GLUT1 expression in the whole organism. To do so, we generated mice that were transgenic for a genomic fragment harboring the human *SLC2A1* locus. Serendipitously, one of the resulting lines was found to contain a truncated fragment harboring only part of the *SLC2A1* gene but the entirety of the *SLC2A1-DT* NAT (Supplemental Figure 3A). PCR analysis of material from brain tissue isolated from mice from this line demonstrated that the NAT was indeed expressed in the line (Figure 2A and Supplemental Figure 3B). Interestingly, this resulted in an increase in murine *Slc2a1* (Figure 2B and Supplemental Figure 3C), suggesting modulatory function of the NAT across species. To determine if the NAT-mediated increase in murine *Slc2a1* was of physiological consequence, we introduced the transgene bearing *SLC2A1-DT* into a well-established mouse model of GLUT1DS, which is haploinsufficient for *Slc2a1* (*Slc2a1^+/–^*) (3). GLUT1-deficient mice harboring the transgene (*Slc2a1^+/–^;SLC2A1-DT^tg^*) and relevant controls were then assessed 3 different ways. In a test of motor activity on a rotarod, we found that *Slc2a1^+/–^;SLC2A1-DT^tg^* mutants performed significantly better than mutants absent the lncRNA but less well than WT controls (*Slc2a1^+/+^*) (Figure 2C). Consistent with the improved motor performance, we found that hypoglycorrhachia (low cerebrospinal fluid [CSF] glucose), a signature feature of GLUT1DS, was mitigated in *Slc2a1^+/–^;SLC2A1-DT^tg^* mutants (Figure 2D). As blood glucose levels in these mutants were unchanged by expression of the transgene, CSF-to-blood glucose ratios were also significantly enhanced (Figure 2E). Hypolactorrhachia (low CSF lactate), an additional characteristic of GLUT1DS (7) was also ameliorated in *Slc2a1^+/–^;SLC2A1-DT^tg^* mutants, with lactate levels in the mice, in these experiments, raised to those observed in WT controls (Supplemental Figure 3D). This suggests that GLUT1 induction by the lncRNA not only raises CSF glucose but also restores metabolic products of glucose. GLUT1DS is characterized by debilitating seizures. In model mice, this phenotype manifests as abnormal spike-wave discharges (SWDs) observed in electro-encephalograms (EEGs) (7, 18). We found that the number of SWDs measured over 24 hours in mutants expressing *SLC2A1-DT* was reduced relative to cohorts devoid of the NAT and no different from those in WT controls (Figure 2F).

Considering elevation of GLUT1 by the *SLC2A1-DT* lncRNA, we inquired if, in addition to its transcription-boosting property (Supplemental Figure 1J), the NAT also affected *Slc2a1* transcript stability. For this, we isolated mouse embryonic fibroblasts (MEFs) from *Slc2a1^+/+^;SLC2A1-DT^tg^* transgenic mice on the one hand and from *Slc2a1^+/+^* littermates on the other and used the cells to assess how quickly *Slc2a1* RNA turned over in the presence or absence of the lncRNA. We found that degradation of the *Slc2a1* transcript was slowed significantly in *Slc2a1^+/+^;SLC2A1-DT^tg^* MEFs when compared with the turnover of the transcript in WT cells (Figure 2G). Our collective findings suggest that (a) the ability of the *SLC2A1-DT* lncRNA to modulate *Slc2a1* expression in cultured cells is maintained in the intact organism, in this instance, a rodent; (b) it is indeed able to function in trans, as the transgene expressing the lncRNA was mapped to an altogether different region of the mouse genome (Chr.12qC2) from mouse *Slc2a1* (Chr.4qD2.1); (c) the ability of the lncRNA to raise *Slc2a1* expression is of physiological consequence, reflected in amelioration of the GLUT1DS phenotype of model mice; and (d) the lncRNA augments GLUT1 levels, at least in part, by stabilizing the *Slc2a1* transcript.

### SLC2A1-DT from an exogenous source mitigates overt disease in GLUT1DS-model mice.

**Figure 3:**
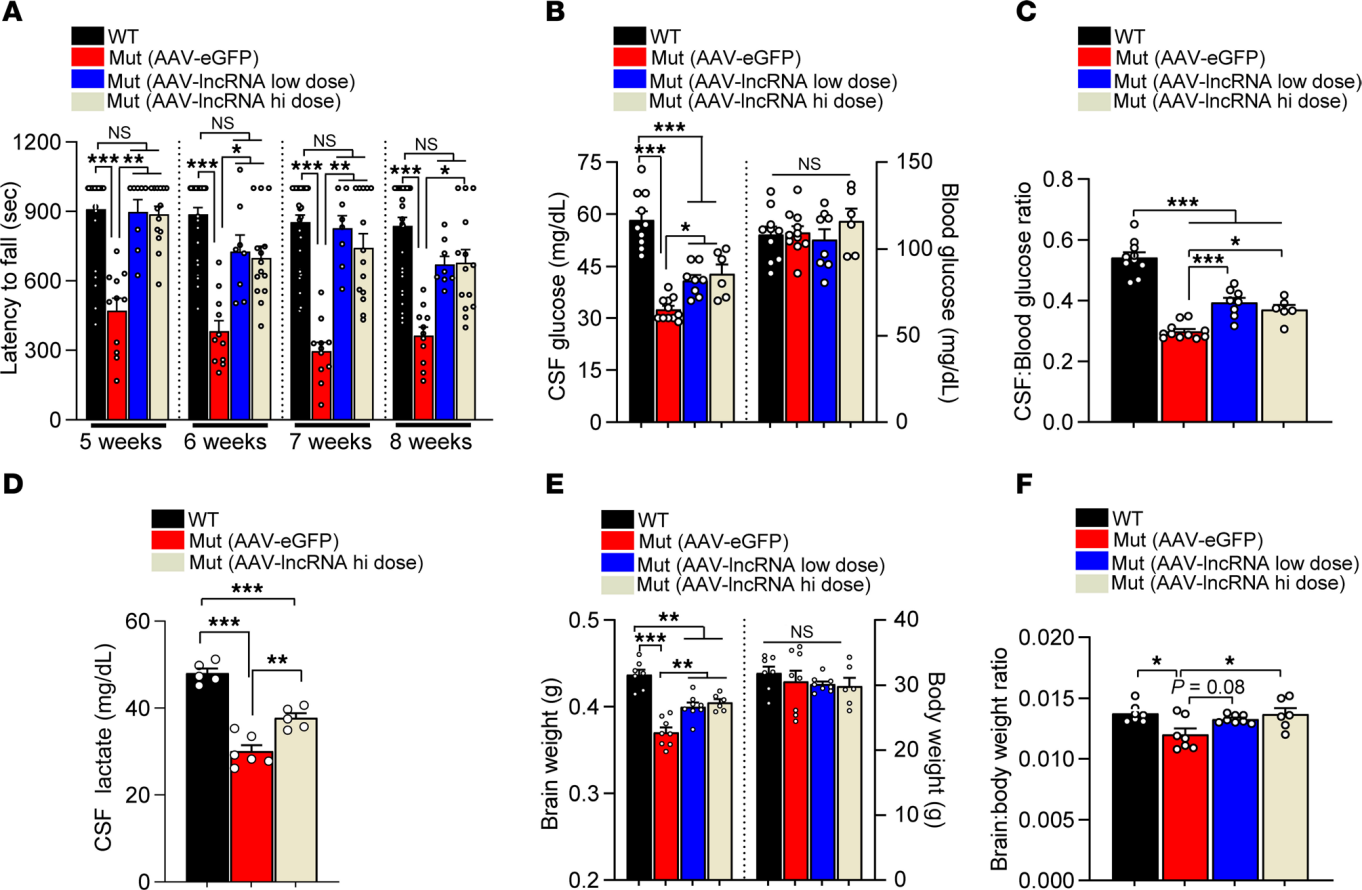
Viral vector-mediated delivery of the *SLC2A1-DT* lncRNA lessens GLUT1DS disease severity in model mice. (**A**) Quantified results of rotarod motor performance tests of the indicated mouse cohorts over a 1-month time period. Note significant improvement in the performance of mutant mice administered the therapeutic lncRNA. *P < 0.05, **P < 0.01, ***P < 0.001, Kruskal-Wallis test, *n* = 32, 11, 8, and 15 for WT, AAV-eGFP–dosed, AAV-lncRNA–low dosed, and AAV-lncRNA–high dosed mice, respectively. Graphs depicting (**B**) CSF and blood glucose levels and, (**C**) CSF-to-blood glucose ratios in the various cohorts of mice. *P < 0.05, ***P < 0.001, 1-way ANOVA, *n* = 10, 11, 8, and 6 for WT, AAV-eGFP–dosed, AAV-lncRNA–low dosed, and AAV-lncRNA–high dosed mice, respectively. (**D**) CSF lactate levels in control and mutant mice treated with the lncRNA or eGFP construct. **P < 0.01, ***P < 0.001, 1-way ANOVA, *n* = 5 each for WT and AAV-lncRNA–dosed mice and *n* = 6 for, AAV-eGFP–dosed mice. (**E** and **F**) Graphs showing evidence of reduced micrencephaly in GLUT1DS mutant mice administered either low or high dose of AAV-lncRNA. *P < 0.05, **P < 0.01, ***P < 0.001, 1-way ANOVA, *n* = 7, 8, 8 and 6 for WT, AAV-eGFP–dosed, AAV-lncRNA–low dosed and AAV-lncRNA–high dosed mice, respectively. Outcomes quantified in **B**–**F** were assessed in 5-month-old mice.

Considering its effects when expressed as a transgene and given our quest to develop what we believe would be novel GLUT1DS therapies that address the root cause — low GLUT1 — of the condition, we inquired if *SLC2A1-DT* from an exogenous source might also mitigate disease in model mice. To test this possibility, we packaged the lncRNA into a self-complementing adeno-associated viral vector (scAAV.PHP.eB) (19) and delivered it systemically at 2 different concentrations, 4.2 × 10^11^ VGs (low dose) and 8.4 × 10^11^ VGs (high dose), to postnatal 1 (PND1) GLUT1DS mutant mice. We began by assessing the effects of such delivery on motor performance of 5-week-old lncRNA-treated mutant mice. AAV-eGFP-treated mutants and WT *Slc2a1^+/+^* mice served as controls. As expected, AAV-eGFP–treated mutants performed very poorly relative to WT controls on the rotarod. In contrast, the performance of mutants delivered either a low or high dose of the AAV-lncRNA was indistinguishable from that of *Slc2a1^+/+^* mice (Figure 3A). Notably, this outcome was sustained over a 4-week period despite a subtle overall decrease in latency to fall off the rotarod in all mouse cohorts. Differences between the mutants treated with the low or high doses of the lncRNA were mostly undetectable. We next assessed the effect of the lncRNA on hypoglycorrhachia. Measurements of CSF glucose revealed that, while levels in lncRNA-treated mutants were lower than those in WT mice, they were nevertheless significantly higher than those of AAV-eGFP–treated mutants (Figure 3B). Akin to observations in our transgenic lines, blood glucose levels remained unaltered whereas CSF-to-blood glucose ratios rose in lncRNA-treated versus eGFP-treated mutants (Figure 3, B and C). Curious to determine if the increase in CSF glucose in the AAV-lncRNA–treated mutants also raised CSF lactate, and considering similar outcomes in animals treated with the 2 different lncRNA doses, we proceeded to quantify lactate concentrations in controls and a cohort of mutants injected with the high dose of our therapeutic vector. Consistent with CSF glucose measurements, we found that CSF lactate had been augmented significantly in AAV-lncRNA mutants relative to lactate levels in AAV-eGFP–treated mice, although they remained lower than levels in healthy controls (Figure 3D). GLUT1DS patients exhibit decelerating head growth (7). In model mice, this manifests as micrencephaly (3). Expectedly, brain sizes of AAV-eGFP–treated *Glut^+/–^* mutants were markedly smaller than those of *Slc2a1^+/+^* controls, notwithstanding equivalent body weights. In contrast, and consonant with previous outcomes, the micrencephalic phenotype was significantly less severe in mutants treated with AAV-lncRNA (Figure 3, E and F). Differences between the cohorts treated with low- or high-lncRNA doses were once again undiscernible. These results suggest that *SLC2A1-DT* from an exogenous source is indeed salutary to GLUT1DS mutants. Moreover, our inability to detect differences between mutants administered high or low doses of AAV-lncRNA suggested that the NAT has a ceiling effect. This ceiling appears to be reached, in the rodent model, with the low dose of the therapeutic vector.

### AAV-mediated delivery of SLC2A1-DT raises GLUT1 and ameliorates brain pathology in GLUT1DS mutant mice.

**Figure 4:**
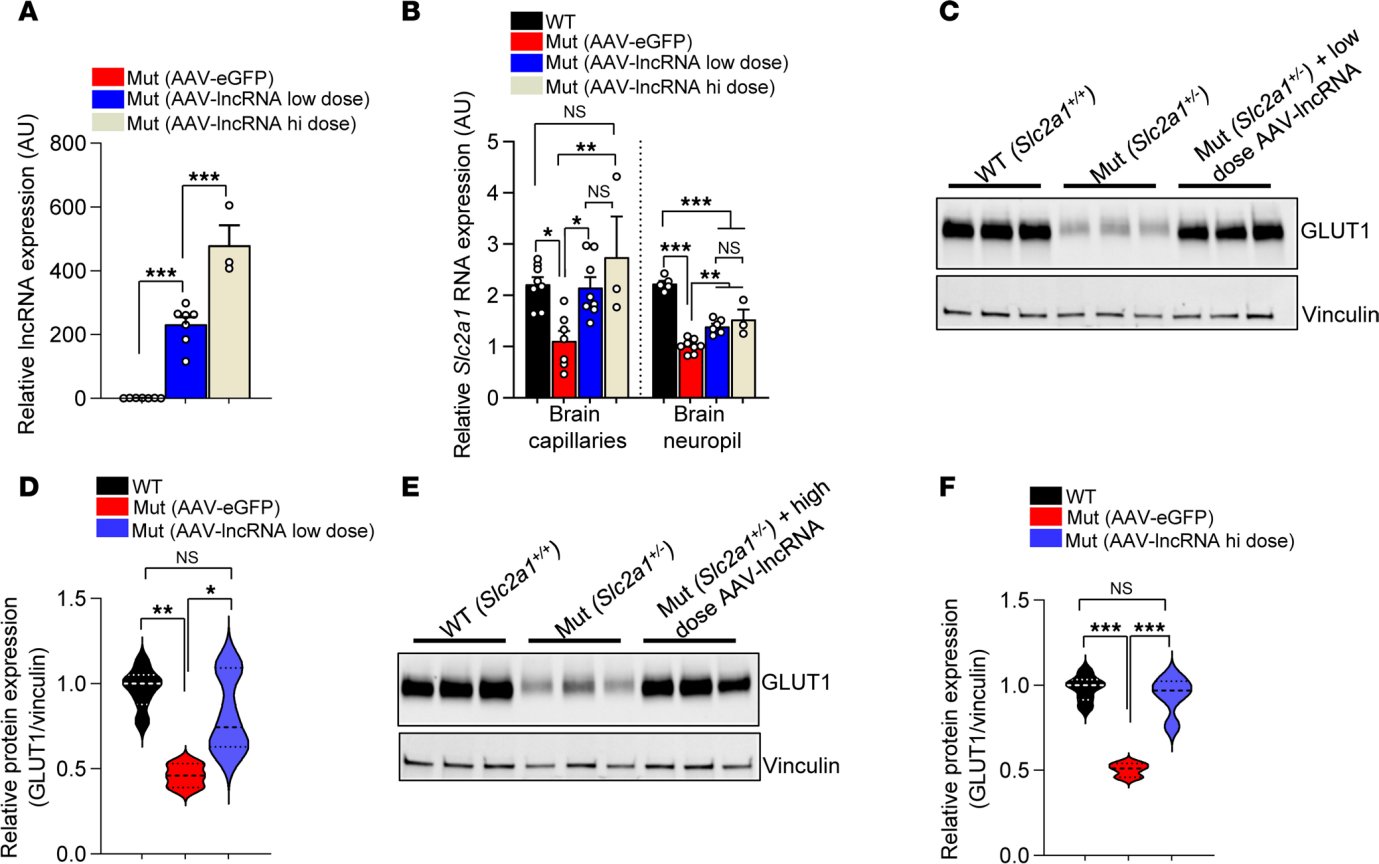
AAV-mediated delivery of the *SLC2A1-DT* lncRNA stimulates GLUT1 expression in GLUT1DS-model mice. (**A**) Results of Q-PCR on brain tissue of mice showing robust expression of the lncRNA in GLUT1DS mutants administered either a low or high dose of AAV-lncRNA. ***P < 0.001, one-way ANOVA, *n* = 7 each for AAV-lncRNA–low dosed and AAV-eGFP–dosed mice and *n* = 3 for, AAV-lncRNA–high dosed mice. (**B**) AAV-mediated expression of the lncRNA raises murine *Slc2a1* RNA levels in brain fractions of the 2 relevant cohorts of mutants in comparison with mutants administered an AAV-eGFP construct. *P < 0.05, **P < 0.01, ***P < 0.001, 1-way ANOVA, *n* = 8, 7, 8 and 3 for WT, AAV-eGFP–dosed, AAV-lncRNA–low dosed and AAV-lncRNA–high dosed mice, respectively. (**C**) Representative Western blot and (**D**) quantified representation of murine GLUT1 protein in brain blood vessels of mutants administered the low dose of AAV-lncRNA and relevant controls. *P < 0.05, **P < 0.01, 1-way ANOVA, *n* = 6 for WT and AAV-lncRNA–low dosed mutant mice, and *n* = 3 for AAV-eGFP–dosed mice. (**E**) Representative Western blot and (**F**) graphical plot of murine GLUT1 protein in cerebral blood vessels of mutant mice administered the high dose of AAV-lncRNA and relevant controls. ***P < 0.001, 1-way ANOVA, *n* = 6 for WT and AAV-lncRNA–high dosed mutants, and *n* = 3 for AAV-eGFP–dose mice.

To investigate the molecular basis of disease rescue in mutant mice administered the lncRNA, we examined levels of the transcript in the brain microvasculature fractions of these mice and controls administered the AAV-eGFP construct. Q-PCR analysis revealed robust expression of the lncRNA in mutant mice administered the low dose of the vector and even greater transcript levels in mutant mice injected with the high dose of the vector (Figure 4A). Expectedly, AAV-eGFP-injected animals expressed negligible amounts of the human lncRNA. Next, we assessed the effect of the lncRNA on *Slc2a1* RNA in the various cohorts of mice. Predictably, AAV-eGFP–injected mutants expressed significantly lower GLUT1 in brain capillary and neuropil fractions relative to amounts of the transcript in these compartments of WT control mice (Figure 4B). In contrast, but consistent with lncRNA measurements, *Slc2a1* transcript was raised in the 2 sets of mutant mice injected with AAV-lncRNA. Interestingly, GLUT1 in brain capillaries but not neuropil of these mutant mice was completely normalized, suggesting a more potent effect of the lncRNA in tissue known to express abundant GLUT1 (20). Reflective of a ceiling effect of the lncRNA, GLUT1 levels were equivalent in mutants administered low or high dose of the vector in each of the brain fractions examined. Western blots to assess GLUT1 protein in brain tissue of the various cohorts confirmed the RNA expression analysis; the lncRNA restored levels of the transporter to mutants administered the different doses of therapeutic vector (Figure 4, C–F).

**Figure 5:**
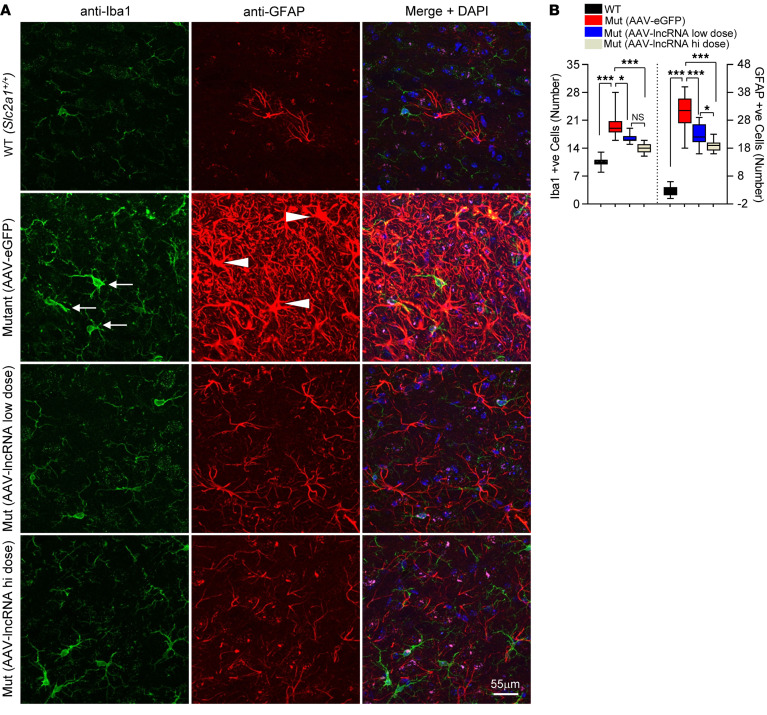
AAV-mediated delivery of the *SLC2A1-DT* lncRNA reduces neuroinflammation in GLUT1DS-model mice. (**A**) Representative thalamic sections from 4–5-month-old control and mutant mice treated with the lncRNA. Pronounced neuroinflammation in AAV-eGFP-treated mutant mouse characterized by activated, hypertrophic microglia (arrows), and reactive astrocytes (arrowheads) is observed. Fewer such cells were observed following treatment with AAV-lncRNA. (**B**) Graph depicts quantified numbers of reactive astrocytes and activated microglia in the various cohorts of mice. *P < 0.05, ***P < 0.001, Kruskal-Wallis test (microglia) and 1-way ANOVA (astrocytes), *n* = 3 nonadjacent fields from *N* = 6, 3, 5 and 5 for WT, AAV-eGFP-dosed, AAV-lncRNA-low dosed and AAV-lncRNA-high dosed mice, respectively.

**Figure 6:**
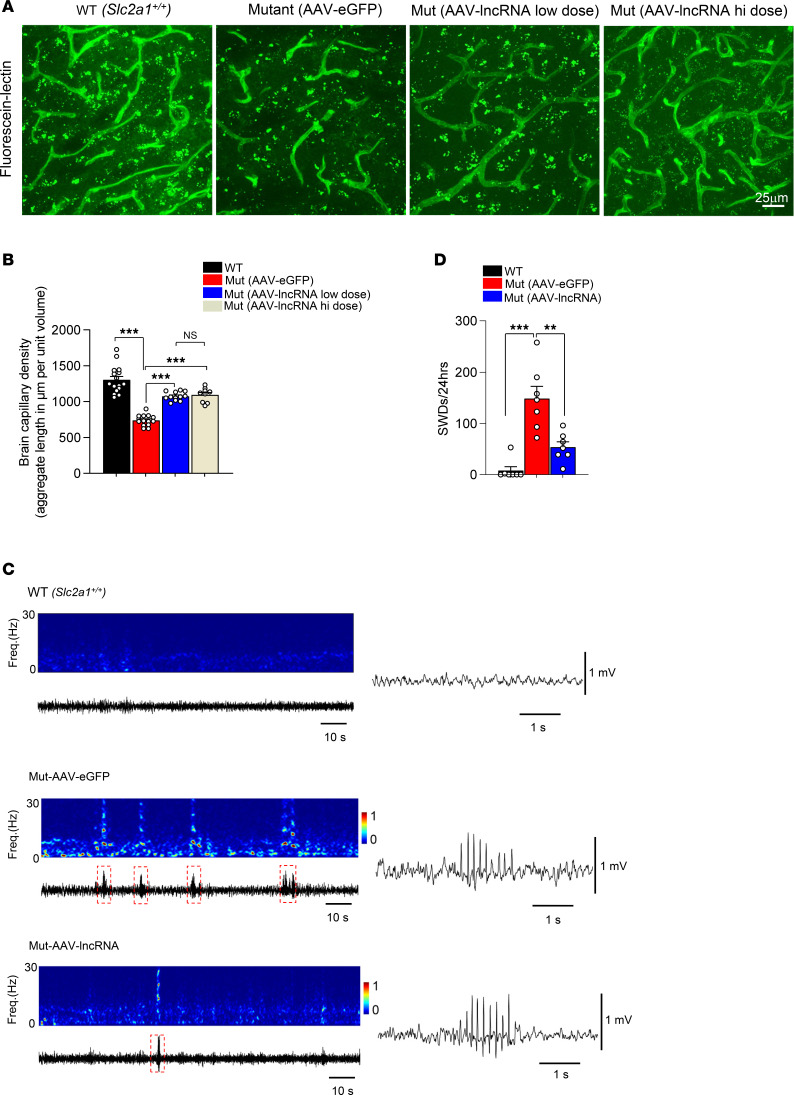
AAV-mediated delivery of the *SLC2A1-DT* lncRNA stimulates brain capillary formation and reduces seizures in GLUT1DS model mice. (**A**) Representative thalamic sections from 4–5-month-old control and mutant mice treated with the lncRNA. Sections were stained with labeled lectin to reveal brain capillaries. Note fewer capillaries in mutant mouse treated with AAV-eGFP and a relative normalization of the microvasculature in mutant mouse treated with AAV-lncRNA. Scale bar: 25 μm.(**B**) Graph quantifies aggregate cerebral capillary length in the mice. ***P < 0.001, 1-way ANOVA, *n* = 9 regions from each of *N* = 6, 3, 5 and 5 for WT, AAV-eGFP–dosed, AAV-lncRNA–low dosed and AAV-lncRNA–high dosed mice, respectively. (**C**) Representative EEG spectrograms at 0–30Hz (colored panels) and corresponding traces (below the colored panels) depicting individual SWDs, highlighted in red boxed areas, captured over 2 minutes or 5 seconds, in order to emphasize seizure activity, in the various cohorts of mice. (**D**) Quantification of SWDs in the 3 cohorts of mice. **P < 0.01, ***P < 0.001, Mann-Whitney tests, *n* = 7 mice in each group.

We previously showed that GLUT1 haploinsufficiency in model mice triggers profound and early neuroinflammation (21). This is accompanied by arrested development of the brain microvasculature (18, 21). Accordingly, we examined brain sections of our mice immunohistochemically to investigate the effects of the lncRNA on these pathological aspects of the disease. Consistent with our prior studies, thalamic sections from mutants treated with the control vector continued to exhibit a clearly discernible neuroinflammatory response characterized by reactive astrocytes and activated microglia (Figure 5, A and B, and Supplemental Figure 4). These hypertrophied glial cells were mostly absent in WT controls and significantly reduced in numbers in mutants administered the therapeutic vector. Similar brain sections stained with labeled lectin to highlight brain capillaries revealed a diminutive brain microvasculature in mutants treated with AAV-eGFP. In comparison, mutants treated with the therapeutic vector had significantly greater densities of capillaries (Figure 6, A and B, and Supplemental Figure 5A), although the extent of the brain microvasculature did not reach the WT state. In a final assessment of the effect of the lncRNA on brain pathology, we carried out EEGs on mutants administered the low dose of therapeutic vector and compared the incidence of SWDs with those in the 2 control cohorts. Consistent with observations made in experiments carried out on our transgenic mice, we found that seizures in AAV-lncRNA-treated *Slc2a1^+/–^* mutant mice were reduced (Figure 6, C and D). Moreover, whereas mice treated with the AAV-eGFP construct not only exhibited significantly greater numbers of SWDs but also the occasional convulsive seizure (Supplemental Figure 5B), this latter type of seizure was completely suppressed in mutant mice injected with the lncRNA-containing construct. These findings are consistent with other outcomes examined in mutant mice treated with AAV-lncRNA and constitute compelling evidence of the therapeutic effects of delivering this modulatory RNA in a viral vector to a model of GLUT1DS.

### Sustained therapeutic effects of AAV-mediated delivery of SLC2A1-DT to GLUT1DS-model mice.

**Figure 7:**
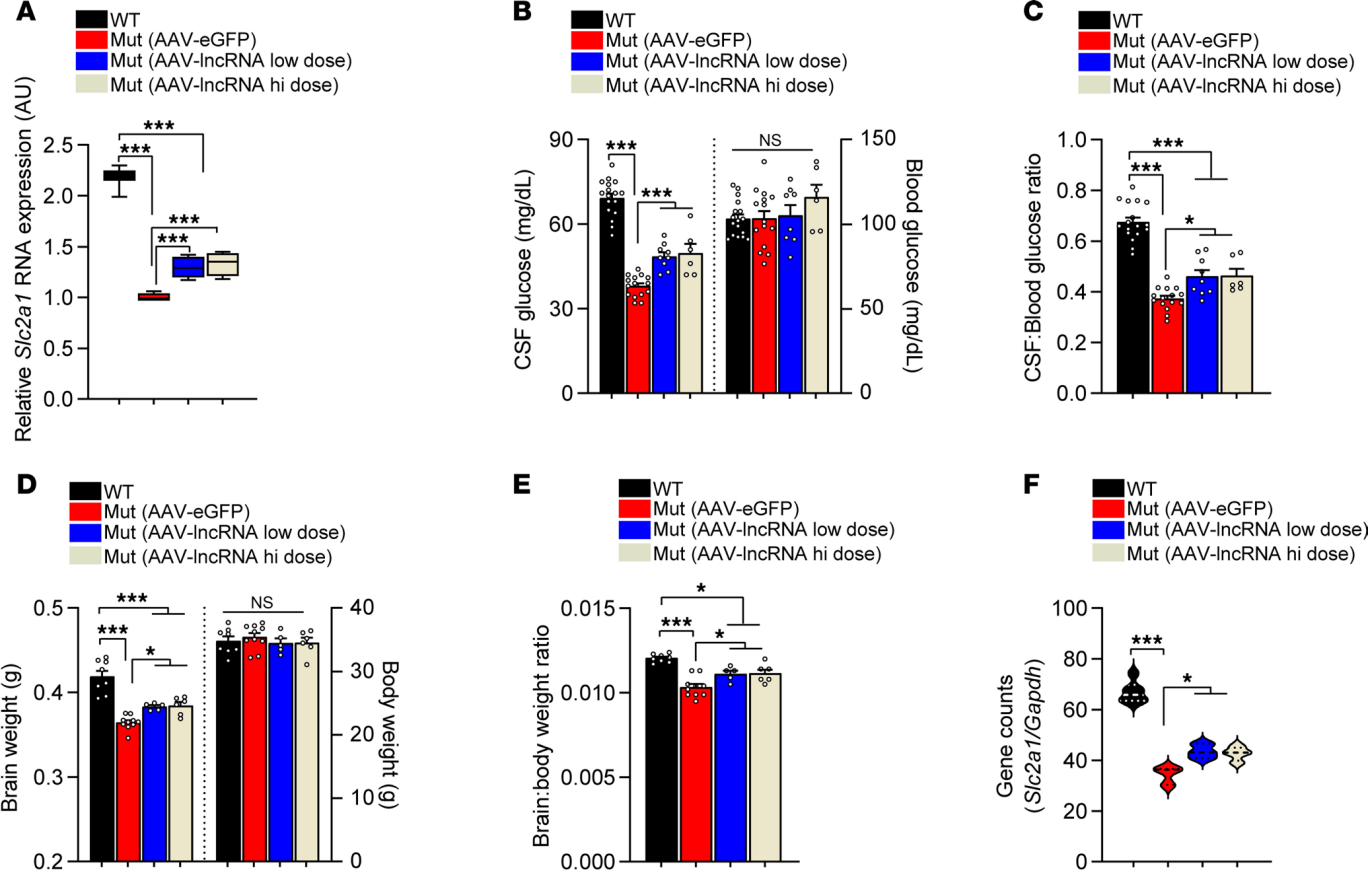
Sustained therapeutic effects deriving from AAV-mediated delivery of the *SLC2A1-DT* lncRNA. (**A**) *Slc2a1* expression in brain tissue of GLUT1DS mutants treated with the AAV-lncRNA vector remains significantly higher than it is in mutants treated with the AAV-eGFP construct. ***P < 0.001, 1-way ANOVA, *n* = 9, 6, 6 and 4 for WT, AAV-eGFP–dosed, AAV-lncRNA–low dosed, and AAV-lncRNA–high dosed mice, respectively. (**B**) Quantified levels of CSF and blood glucose levels depict significantly higher levels of the former in mutant mice treated with the AAV-lncRNA vector relative to concentrations in mutants treated with the AAV-eGFP control vector. Blood glucose levels were equivalent in all 4 mouse cohorts. ***P < 0.001, 1-way ANOVA, *n* = 17, 15, 9 and 6 for WT, AAV-eGFP–dosed, AAV-lncRNA–low dosed and AAV-lncRNA–high dosed mice, respectively. (**C**) Quantified CSF-to-blood glucose ratios reflect higher levels of CSF glucose levels in AAV-lncRNA–treated mutants vis-à-vis AAV-eGFP-treated–model mice. *P < 0.05, ***P < 0.001, 1-way ANOVA, sample sizes equivalent to those in **B**. The micrencephalic phenotype remains less severe in GLUT1DS mutants expressing the lncRNA compared with the condition in mutant mice injected with the AAV-eGFP vector, as assessed by measuring (**D**) brain and body sizes and (**E**) calculating brain-to-body weight ratios. *P < 0.05, ***P < 0.001, 1-way ANOVA, *n* = 8, 10, 5, and 6 for WT, AAV-eGFP–dosed, AAV-lncRNA–low dosed and AAV-lncRNA–high dosed mice, respectively. (**F**) Gene counts from RNA-Seq analysis of the various cohorts of mice illustrate sustained higher expression of *Slc2a1* in mutants treated with AAV-lncRNA compared with mutants treated with the control AAV. *P < 0.05, ***P < 0.001, 1-way ANOVA, *n* = 6 (WT), *n* = 3 (vehicle-treated and low-dose lncRNA-treated) and *n* = 4 (AAV-lncRNA, high-dose treatment).

Durability of benefit that is accrued following gene delivery in a viral vector is critically important for its future use in the clinic. Accordingly, a subset of the various cohorts of mice generated for our experiments and treated with therapeutic or control vector were allowed to age. Twelve months following treatment, these mice were examined for evidence of sustained benefit from expression of the lncRNA. We began by examining GLUT1 levels in the mice. As expected, AAV-eGFP–treated mutants continued to express low GLUT1 in whole-brain tissue relative to WT controls. In contrast, the comparatively higher levels of GLUT1 we’d observed in mutants delivered either low or high doses of the lncRNA persisted in the 12-month-old cohorts. These animals continued to express approximately 30% more *Slc2a1* transcript than did mutants administered the control AAV (Figure 7A), which is a likely consequence of sustained lncRNA activity from episomally maintained transcript delivered to cells by the AAV. We next assessed CSF glucose levels in the different mice. Consistent with higher brain GLUT1 levels, we found that mutant mice expressing the lncRNA continued to exhibit greater concentrations of CSF glucose than those in mutant mice administered the AAV-eGFP construct; blood glucose levels remained unaltered (Figure 7B). The combination of higher CSF glucose levels and unchanged blood glucose concentrations resulted in enhanced CSF-to-blood glucose ratios in the AAV-lncRNA versus AAV-eGFP–treated mutant mice (Figure 7C). In a final set of assessments to determine persistence of therapeutic effect of *SLC2A1-DT*, we examined brain size in the treated mice. The micrencephalic phenotype in mutant mice treated with AAV-lncRNA remained less severe than it was in AAV-eGFP-treated mutant mice (Figure 7D). Body weights in the various cohorts of mice were statistically indistinguishable but, consonant with measurements of brain size, brain-to-body weight ratios were higher in AAV-lncRNA versus AAV-eGFP–treated mutants (Figure 7, D and E). Together, these results attest to the durability of the effect of the lncRNA delivered to GLUT1DS model mice in a viral vector.

In anticipation of developing the lncRNA into a clinical therapeutic for GLUT1DS or other conditions characterized by low GLUT1 protein, and to gain insight into its mode of action, we surveilled the transcriptomes of brain tissue from 12-month-old GLUT1DS mutant mice administered AAV-lncRNA as pups. The resulting RNA-Seq data was then analyzed for toxicological processes using the Tox Analysis function of Qiagen’s Ingenuity Pathway Analysis (IPA) tool. This indicated that the expression of the lncRNA is not significantly associated with any toxicity. Moreover, RNA-Seq analysis of WT mice and mutant mice administered either dose of the lncRNA revealed a total of 44 significantly differentially expressed transcripts between the mutants and controls (Supplemental Table 1). A similar comparison of the transcriptomes of WT mice and mutants treated with AAV-eGFP uncovered 56 differentially expressed genes (DEGs) (Supplemental Table 2). Interestingly, except for *Slc2a1 (Glut1)*, none of the genes that were perturbed in this second analysis were present in the list of DEGs compiled from the comparison of WT animals and lncRNA-treated mutant mice (Supplemental Tables 1 and 2), suggesting that at least a subset of genes that were dysregulated in *Slc2a1^+/–^* mutant mice owing to low GLUT1 were normalized in expression following treatment with the lncRNA. As a specific example, we highlight TXNIP, a known interactor and facilitator of GLUT1 activity (22), which was significantly altered in AAV-eGFP-treated mutant mice but normalized in expression by the lncRNA (Gene counts: WT=26.87 ± 2.54; Mut-AAV-eGFP=18.34 ±0.20; Mut-AAV-lncRNA=25.57 ± 1.34, *P* < 0.05, Mut-AAV-eGFP vs WT and Mut-AAV-eGFP vs Mut-AAV-lncRNA, 1-way ANOVA, *n* = 3–6 mice of each cohort). Moreover, consonant with Q-PCR of *Slc2a1* expression in the various cohorts, the difference in *Slc2a1* transcripts between WT and mutant mice administered the therapeutic vector was smaller than it was when WT mice were compared to AAV-eGFP-treated mutant mice (Figure 7, A and F, and Supplemental Tables 1 and 2). Notably, several DEGs that were revealed by comparing WT and AAV-eGFP-treated mutant mice were also present in the list of 198 DEGs catalogued by comparing mutant mice treated with either lncRNA or the eGFP construct (Supplemental Table 3), suggesting that dysregulation of at least some of these are a result of eGFP overexpression rather than low GLUT1. Gene ontology (GO) analysis of DEGs in WT versus AAV-eGFP-treated mutant mice to identify biological processes perturbed in GLUT1DS revealed several involved in maintaining neuronal health (Table 1). Interestingly, consistent with the GLUT1-inducing effect of *SLC2A1-DT*, similar GO analysis of DEGs in WT versus lncRNA-treated mutants failed to reveal perturbations of any of these processes, suggesting once again, that the lncRNA normalizes processes deranged by low GLUT1. To complement the IPA analysis and ensure that long-term expression of the *SLC2A1-DT* lncRNA is benign, we carried out a histological study of the major organ systems of 12-month-old mutants treated as pups with AAV-lncRNA. A comparison of H&E-stained sections of the various organs from these mutants with those of controls failed to reveal any gross cellular or morphological abnormalities associated with the sustained expression of the lncRNA (Supplemental Figure 6). These results support the IPA analysis and once again suggest that persistent expression of the lncRNA is safe. This raises optimism for future applications of the transcript for clinical therapies involving neuroglycopenia.

## Discussion

Glucose is the mammalian brain’s preferred energy source and is delivered to the neuropil via GLUT1, the principal cerebral glucose transporter. Given the brain’s limited capacity to store glucose, nutrient supply to the organ must be precisely adjusted and the amounts transported into the parenchyma continuously altered to satisfy the brain’s immediate energy requirements. Calibrating GLUT1 activity to respond to these needs is therefore crucial. Here, we report on a little-described natural antisense *SLC2A1* lncRNA transcript engaged in such regulation and examine the therapeutic potential of the transcript for conditions involving perturbed GLUT1 levels. Six principal findings emerge from our study. First and foremost, we show that the lncRNA is unlikely to be a consequence of pervasive genomic transcription, instead having evolved to play a vital role in regulating its sense-strand partner, *SLC2A1*. Unexpectedly, the lncRNA regulates *SLC2A1* in a concordant fashion. Second, and equally notable, are results demonstrating that, notwithstanding its location in immediate proximity to *SLC2A1*, indeed, overlapping part of the protein-coding gene, the lncRNA can produce its effects in trans. A third and related intriguing observation is that the lncRNA regulates GLUT1 across species. In this instance, the human lncRNA stimulated expression of not only the human *SLC2A1* gene but also its murine homolog, attesting to the significance and conserved relationship between the antisense transcript and its protein-coding cognate. A fourth noteworthy finding is that the lncRNA truly appears to tune GLUT1 expression; our evidence suggests that *Slc2a1* stimulation by the lncRNA plateaus and is subject to a ceiling effect. This precludes potential adverse outcomes resulting from overexpressing the transporter. Fifth, we demonstrate that the lncRNA effects GLUT1 increase at least in part by stabilizing the transporter’s transcript. Finally, and perhaps most notably, we show that the lncRNA is of therapeutic value. Using an established mouse model of GLUT1DS, we demonstrate that the lncRNA can induce endogenous *SLC2A1* expression and raise GLUT1 levels sufficiently to mitigate disease. Our results raise the prospect of employing the lncRNA for myriad other conditions, including cancer, diabetes, and AD, in which GLUT1 levels are disrupted.

Glucose homeostasis and therefore GLUT1 activity are critical to the health and viability of the organism (6). This is especially true for the mammalian brain. Consequently, it is unsurprising that GLUT1 expression is subject to intricate control. The role of noncoding transcripts in such control has become evident only relatively recently but is consistent with an emerging appreciation of the overall physiological importance of regulatory RNAs. Reports of lncRNA-mediated control of GLUT1 do exist (23–32). However, most of the associated lncRNAs referenced lie outside the *SLC2A1* locus, and a preponderance was reported to function by antagonizing miRNAs that interact with and modulate *SLC2A1* expression. The specific effects of the NAT we describe here have never been reported. Moreover, in the only study in which *SLC2A1-DT* was described to regulate *SLC2A1* expression, the relationship between sense and antisense genes was, surprisingly, found to be discordant; the lncRNA suppressed *SLC2A1* expression (32). One explanation for the contrast in findings to those reported here is that the previous investigation focused exclusively on hepatic cell lines — tumorigenic and non-tumorigenic — whereas our findings derive from results obtained from multiple mouse and human tissues.

The concordance in expression between *SLC2A1-DT* and *SLC2A1* we report is unexpected, as most NATs described antagonize their sense-strand partners (16). Yet, there are numerous instances of such transcripts stimulating expression of their protein-coding cognates. Noteworthy examples in the context of CNS conditions are *BACE1-AS*, which enhances expression of the b-secretase 1 (*BACE1*) gene implicated in AD (33); *RAB11b-AS1*, which concordantly regulates *RAB11b*, a vesicular trafficking protein linked to autism spectrum disorder (34); and *UCHL1-AS1*, which stimulates *UCHL1* expression, a ubiquitin-hydrolase-encoding Parkinson’s disease susceptibility gene (35). Other established NATs, identified in the context of cancer, that induce their sense-strand partners include *Khsp1* and *ZNF667-AS1* (36, 37). *SLC2A1-DT* and *SLC2A1* add to this list. Importantly, considering a growing interest in the therapeutic value of noncoding RNAs and our quest to address the root cause of GLUT1DS, our results also serve as the basis of developing *SLC2A1-DT* clinically. The physiological relevance of this lncRNA transcript stems from several observations including its ability to elicit an effect on GLUT1 across species and in trans. Consistent with these effects, we note that similarly positioned antisense transcripts have been identified in all mammals surveyed thus far (Supplemental Table 4). Interestingly, birds, as represented by the domestic chicken, are devoid of such a transcript, suggesting that the lncRNA emerged relatively recently in evolution. This is consistent with the larger, more sophisticated mammalian brain and thus the need for a more refined means of catering to its energy requirements.

Despite a growing interest in the therapeutic value of regulatory RNAs (38), and an emerging appreciation of their use as diagnostic markers, e.g., *PCA3* and *DSCAM-AS1* for prostate and breast cancers, respectively (39), we are unaware of RNA therapies that employ lncRNAs. Proof-of-concept studies to stimulate gene expression by NAT-type molecules have been carried out, but these have involved lncRNA mimics rather than the unaltered transcripts (40, 41). In this respect, the work we describe here is, presumably, substantially novel. In addition to directly addressing the underlying cause — low GLUT1 — of GLUT1DS and thus providing an alternative to ketogenic diets for the condition, the therapeutic modality described here has considerable advantages over *SLC2A1* gene replacement, which is prone to trigger GLUT1 overexpression. Such overexpression, which frequently results from AAV-mediated gene delivery, is undesirable, as supraphysiological GLUT1 is an established risk factor in cancers (42, 43). The ceiling effect associated with the *SLC2A1-DT* NAT that limits GLUT1 increase to roughly WT levels resolves this danger. Moreover, use of the lncRNA is expected to stimulate and restore GLUT1 only in tissues that express the endogenous transporter physiologically. This property of the lncRNA precludes off-target effects, a second distinct advantage of using the transcript instead of *SLC2A1* for therapeutic purposes. Notwithstanding these observations, it is recognized that, as with all regulatory RNAs, lncRNAs as therapeutic molecules are not without risk. Off-target effects are the most obvious and may not be fully appreciated in the rodent model. The prospect of *SLC2A1-DT* NAT raising levels of mutant GLUT1 proteins in patients harboring missense *SLC2A1* mutations is also real, even if it is unclear if and how such mutant proteins might disrupt transporter activity. Ongoing and future studies aim to address these caveats.

While our study has demonstrated the therapeutic value of the *SLC2A1-DT* NAT and clearly shown that the transcript raises GLUT1 expression, it has only partially evaluated how the NAT effects gene induction. Still, some predictions may be made from the existing literature and our own observations. Natural antisense transcripts frequently modulate transcriptional activity by altering chromatin structure. It would therefore not surprise us if such a mechanism underlies the effect of *SLC2A1-DT* on GLUT1 expression. The ability of the NAT to enhance expression of a reporter driven by a *SLC2A1* promoter fragment (Supplemental Figure 1J) hints at this means of gene induction. Considering this outcome, our results, demonstrating a second mechanism of action, i.e., stabilization of the *SLC2A1* transcript by the NAT, was unexpected; it is uncommon for the same regulatory factor to modulate genes via disparate mechanisms. Still, such factors do exist and have been described. For instance, in separate work from our laboratory, we have demonstrated that a Hspa8^G470R^ synaptic chaperone variant modulates the expression of the Survival Motor Neuron 2 (*SMN2*) gene not only by serving as a splice modulator but also by stabilizing the SMN protein (44). Of even greater relevance to findings made here is a report focusing on the Apolipoprotein Antisense1 (*APOA1-AS1*) NAT. This NAT has been reported to not only enhance transcription from its protein-coding *APOA1* cognate by altering the chromatin in the promoter and enhancer of the gene (45) but also to recruit the TATA-box binding protein associated factor 15 (TAF15) protein thereby stabilizing and augmenting concentrations of the SMAD family member 3 (*SMAD3*) mRNA (46). The TRAF3-interacting protein 2 antisense 1 (*TRAF3IP2-AS1*) lncRNA is a second NAT with dual mechanisms of action. On the one hand, it binds the poly (ADP-ribose) polymerase 1 (PARP1) mRNA, accelerating the latter’s decay. On the other, it acts as a tumor suppressor, sequestering miR-200a-3p/153-3p/141-3p and preventing the miRNAs from degrading the tumor suppressor, Phosphatase and tensin homolog (PTEN) transcript (47). Dual control of *SLC2A1* by *SLC2A1-DT* to promote transcription on the one hand and stabilize *SLC2A1* transcript on the other would therefore not be unprecedented. Still, the identities of the chromatin modifiers that might be operating in conjunction with *SLC2A1-DT* at the *SLC2A1* promoter remain to be determined, as do factors potentially involved in stabilizing *SLC2A1* transcript by the lncRNA. Notwithstanding these caveats, which will be addressed in future research, this study has not only presented a potential clinical alternative to the ketogenic diet for GLUT1DS but also done so via an uncommon therapeutic modality: the direct use of a natural antisense transcript.

## Methods

### Sex as a biological variable.

Except for assessing micrencephaly, which is confounded by mixing sexes and is only reported for male mice, our experiments did not discriminate between males and females. Note that the trends reported for the micrencephalic phenotype in male animals are similar to those observed in females in our laboratory.

### Primary cell cultures and assessments of human tissue.

Human fibroblasts were cultured in M-106 medium (Life Technologies) supplemented with Low Serum Growth Supplement (LSGS, Cat. # S-003-10), 10% fetal bovine serum (Gibco), and 1% penicillin-streptomycin-glutamine (Fisher Scientific Inc.). Human primary brain microvascular endothelial cells (BECs) were acquired from Cell Biologics (Cat. # H-6023) and grown in complete human endothelial cell medium (Cat. # H1168). To knockdown or overexpress the *SLC2A1-DT* lncRNA, fibroblasts or BECs were seeded in 6-well plates and grown to ~70% confluency before being transfected. Over-expression of the lncRNA was accomplished by electroporating cells (Nucleofector kit - Cat. # VPD-1001 for fibroblasts; Cat. # VPB-1003 for BECs) with the full-length transcript or truncated versions cloned into the pcDNA3.1 plasmid (Invitrogen). *SLC2A1-DT* knockdown was effected using siRNAs transfected into cells with Lipofectamine 3000 (Invitrogen) according to the manufacturer’s instructions. Seventy-two hours following transfection, all cells were harvested for analysis. Human tissues were obtained from the New York brain bank. Brain tumor tissue was obtained from surgical procedures carried out in the Columbia University department of neurological surgery and banked under IRB AAAJ6163. For the *Slc2a1* RNA stability assay, MEFs were obtained from E12.5 embryos of either WT *(Slc2a1^+/+^)* or *SLC2A1-DT^Tg/Tg^;Slc2a1^+/+^* genotypes. MEFs were cultured in DMEM supplemented with 10% FBS, 100 units/ml penicillin, and 100 μg/ml streptomycin, and maintained at 37°C in a humidified atmosphere containing 5% CO_2_. To determine the half life of the *Slc2a1* mRNA, cells were treated with 7 μg/ml Actinomycin D (Sigma-Aldrich #A9415) for the indicated time points, followed by RNA isolation, cDNA synthesis and Q-PCR (also see SI).

### Gene reporter assays.

A 6 kb region upstream of the *SLC2A1* translational start site was cloned into pGL4.10[luc2] vector (Promega) enabling us to set baseline promoter activity. Effects of the lncRNA on baseline SLC2A1 promoter activity was assessed in HEK293 cells by co-transfecting pGL4.1-6kb-SLC2A1 (2.5mg) with a construct containing the full-length NAT in pcDNA3.1 (2.5mg). Assessments were conducted in triplicate and a pSV-βGal (Promega Inc.) used to normalize transfection efficiency. Seventy-two hours following transfection, cells were lysed in reporter lysis buffer (Promega) and analyzed for luciferase activity (BrightGlo luciferase, Promega Inc.). Luminescence was measured on a GloMax 20/20 Luminometer (Promega). To assess βGal activity, the cell lysate was incubated in Beta-Glo Reagent (Promega Inc.) at RT for 30 minutes and luminescence measured as described above.

### FISH analysis.

Localization of *SLC2A1-DT* transcripts was determined using the QuantiGene ViewRNA ISH Cell Assay Kit (QVC0001, Affymetrix). Briefly, cells were fixed (4% PFA in PBS, 30min. at RT), permeabilized with detergent solution (5 minutes at RT), and then digested with working protease solution (10 minutes at RT). Cells were incubated with a target-specific probe for 3 hours, whereas pre-amplifiers, amplifiers, and label probes were incubated for 30 minutes each. All hybridization steps were carried out at 40°C. Following hybridization, cells were stained with DAPI solution (1 minute at RT) and a drop of antifade (Prolong Gold Antifade Reagent) added to the slide before capturing images on a LEICA TCS SP8 confocal microscope.

### Model mice.

Haploinsufficient *Slc2a1^+/–^* model mice were generated at Columbia University and have been previously described (3). *SLC2A1-DT* transgenic mice were generated for this study by the Columbia University Genetically Modified Mouse Models Core Facility. Briefly, BAC RP11-125O1 (BACPAC Genomics) containing the human *SLC2A1* locus was digested with Cla1 and a 79kb fragment harboring *SLC2A1-DT* isolated and introduced into fertilized mouse oocytes. Resulting *SLC2A1-DT^Tg^* mice were interbred with *Slc2a1^+/–^* animals to produce *Slc2a1^+/–^;SLC2A1-DT^Tg^* cohorts. Experimental mice were generated by breeding mutant male mice with WT females. All mice were maintained on a 129 S6/SvEvTac genetic background and mutants identified for experiments by PCR. Littermate controls were used in all experiments.

### Viral vector production and administration.

Recombinant scAAV.PhP.eB vectors were produced at the Horae Gene Therapy Core, University of Massachusetts as previously detailed (18, 48). Expression of the lncRNA or eGFP cassettes was driven by a CMV-enhanced chicken-b-actin promoter. Vectors were delivered to PND1 pups at concentrations indicated in the Results section via the retro-orbital sinus.

### Phenotypic evaluations and CSF/blood glucose, lactate measurements.

For behavioral outcomes, GraphPad Prism was used to determine sample sizes to detect differences of at least 2 standard deviations with a power of 80% (*P* < 0.05). Mice were not randomized, but as mutants do not exhibit an overt disease phenotype, it was possible to blind the investigator to the specific cohort being assessed. Motor performance was assessed on an accelerating rotarod (Ugo Basile Inc., Italy) as described in the supplemental information (SI) section. Prior to assessing brain and body size, and investigating CSF/serum glucose levels, mice were fasted overnight. Subsequently they were weighed, blood collected from the tail vein, CSF extracted from the cisterna magna as detailed by us in prior studies (18, 21) and the brain removed and weighed. Glucose concentrations in the CSF and blood were assessed using disposable strips and a Contour Next EZ glucose meter (Bayer Corp.). CSF lactate was measured on an Analox GL5 Analyzer (Analox Instruments) using a Lactate II reagent kit (GMRD-093, Analox Instruments).

### Quantitative PCR and western blotting.

*Slc2a1* and *SLC2A1-DT* transcript levels were quantified by Q-PCR (see SI for primers). GLUT1 protein levels in the various cohorts of mice were assessed by western blot analysis using standard techniques as previously described (18, 21). Briefly, cells or tissues were lysed in lysis buffer containing protease inhibitors (Complete Protease Inhibitor Cocktail tablets, Roche, Indianapolis, IA) and 50 μg of total protein resolved by gradient SDS-PAGE (Bio-Rad Labs). Brain parenchymal and vessel fractions were prepared as described in SI. GLUT1 rabbit polyclonal (1:5000; Millipore), SMN mouse monoclonal (1:5000; BD Biosciences) and vinculin rabbit monoclonal (1:5000; Abcam) antibodies were probed with HRP-linked goat anti-rabbit (Invitrogen Inc.) and goat anti-mouse (Jackson Immunoresearch) secondary antibodies each diluted 1:10,000 and visualized on a ChemiDoc Imaging System machine (Bio-Rad Labs) using the ECL Detection Kit (Cat.# 1705061; Bio-Rad Labs). Band intensities were assessed using ImageJ software (NIH).

### Cerebral microvasculature and neuroinflammation.

To examine the cerebral microvasculature, mice were perfused with 1X PBS and then with 4% PFA. Intact brains were then extracted and further incubated overnight in 4% PFA. Coronal sections (50 μm) were cut on a vibratome (LEICA VT1000S) the following day, incubated (1 hour, RT) in blocking solution (3% BSA, 0.5% Triton X-100 in PBS) before further incubating them with fluorescein-conjugated Lycopersicon esculentum lectin. The sections were then washed (3X, 20 minutes) with 1X PBS, mounted with Vectashield (Vector Laboratories) on slides, and overlaid with coverslips for microscopic analysis. Brain capillary density was assessed in 5-month-old mice as previously described (21). Briefly, 20 μm stack images of the thalamus were acquired using a LEICA TCS SP8 confocal microscope. Capillary density in thalamic regions involved quantifying the aggregate length of vessels < 6 μm in diameter in a 184 μm **×** 184 μm area. Three such areas, non-adjacent to one another, in each of at least 4 animals were examined. Images presented are reconstructions of 3D Z-stacks. Note: Total or aggregate length denotes the sum total distance of the stained vessels in the Z-stacks. Gliosis in our mice was examined and quantified in 15 μm stack images of the ventral posteromedial (VPM) thalamic nuclei using antibodies against GFAP and Iba1. Sections were stained and images acquired as described above. Thalamic brain in the region of the VPM nucleus was imaged at a magnification of 20X. Quantification of hypertrophied glial cells was carried out on 63X images. All quantification was carried out by investigators blinded to mouse genotype. Details of antibodies and dilutions employed are supplied in the SI section.

### EEG analysis.

Mice (3-month old) were anesthetized using a ketamine (100mg/kg), xylazine (10mg/kg) mixture administered intra-peritoneally and then placed on a stereotaxic frame with a closed-loop heating system to maintain body temperature. After asepsis, the skull was exposed and a small craniotomy (~0.5mm in diameter) made above the regions of interest. For EEGs, a reference screw was inserted into the skull on top of the cerebellum. EEG recordings were made from 2 screws on top of the cortex 1mm from midline, 1.5mm anterior to the bregma and 1.5mm posterior to the bregma, respectively. The EEG screws were connected to a PCB board which was soldered with a 5-position pin connector. Implants were secured onto the skull with dental cement (Lang Dental Manufacturing). Animals were given a week to recover before being subjected to recordings. EEG recordings were performed for 24–48 hours (light on at 7:00AM and off at 7:00PM) in a behavioral chamber inside a sound attenuating cubicle (Med Associated Inc.). Mice were habituated in the chamber for at least 4 hours before recording began. EEG signals were recorded, bandpass filtered at 0.5–500 Hz, and digitized at 1017 Hz with 32-channel amplifiers (TDT, PZ5 and RZ5D or Neuralynx Digital Lynx 4S). For SWD analysis, FFT of EEG was performed using a 1-second sliding window, sequentially shifted by 0.25-second increments. Then, the “seizure”-power (19–24 Hz) was calculated to extract SWD events based on a threshold of 2–3 standard deviations as described previously (49). A 19–24 Hz band was selected based on its clear separation from normal brain oscillatory activities, although the primary spectral band of SWDs in mice is around 7Hz, which overlaps with theta oscillations during REM sleep or active periods. Two SWD events were merged into one event if their interval was shorter than 1 second. Any SWD event with a duration < 0.5 seconds was removed for analysis. Algorithm-detected SWD events were further reviewed by trained experimenters. Mouse behavior was monitored during recordings using an infrared video camera at 30 frames per second.

### Histology.

H&E staining experiments were performed on paraffin-embedded 8 μm coronal sections (brain) and 4 μm longitudinal sections (liver, heart, kidney, and muscle) and imaged on an Eclipse 80i Nikon microscope (Nikon, Japan) equipped with a DsRi2 camera (Nikon, Japan).

### Mapping transgene insertion site.

To map the insertion site of the transgene harboring the human *SLC2A1* locus in our engineered mice, we used vectorette PCR. Detailed protocols are presented in the SI section of the manuscript and are modified versions of methods used in previous studies (50, 51).

### RNA-seq and IPA analysis.

FASTQ files were aligned to mouse genome assembly GRCm38 using the STAR aligner (52) within the nf-core, rnaseq pipeline (53). Quantification of the aligned data was performed with the default settings of the Salmon tool. After assessing the quality of the data using MultiQC, the resulting counts were analyzed using the limma-voom pipeline (54, 55). Differentially expressed genes (DEGs) were then identified for each pairwise comparison. Note that low and high-dose AAV-lncRNA groups were combined for the purpose of comparisons with control cohorts as we did not observe any significantly altered DEGs between the two lncRNA-treated groups in the RNA-Seq data. To assess for potential toxicity of the lncRNA, we utilized the Tox Analysis function of the Ingenuity Pathway Analysis tool (Qiagen, Inc.), which employs statistical measures to assign z-scores to various toxicological processes predicted to be affected based on the DEGs identified in the RNA-Seq data. In no instance did the analysis show biological process with absolute z-scores greater than 2, which is the cutoff for significant toxicity, for DEGs in the lncRNA-treated mice.

### Gene ontology analysis.

GO analysis was conducted online using the Gene Ontology enrichment analysis & visualization (GORILLA) tool (56). Search parameters were set to identify enriched biological processes at a threshold of *P* < 0.0001. Two unranked lists of genes, a target list of DEGs and a background list of all genes mapped in the RNA-Seq to the Mouse GRCm38.p6 reference genome were queried to identify pathways significantly altered in mutants.

### Statistics.

For data that was normally distributed, the unpaired 2-tailed Student’s *t* test or 1-way ANOVA followed by Tukey’s post hoc comparison, where indicated, were used to compare means for statistical differences. If the data were not found to fit a Gaussian distribution, the Mann-Whitney test or Kruskal-Wallis test followed by Dunn’s multiple comparison test were used to compare ranks. For RNA stability assay, areas under curves (AOCs) were calculated and then compared using the unpaired 2-tailed Student’s *t* test. Data in the manuscript are represented as mean ± SEM. *P* < 0.05 was considered significant. Statistical analyses were performed with GraphPad Prism v9.0 (GraphPad Software).

### Study approval.

All animal procedures adhered to protocols described in the *Guide for the Care & Use of Lab Animals* (National Academic Press, 2011) and were approved by Columbia University’s IACUC. The mice in this study were randomly selected, 129 S6/SvEvTac, male and female mice housed in a controlled environment on a 12-hour light/dark cycle with food and water.

### Data availability.

Underlying values associated with the data presented in the study are provided in the Supporting Data Values file. The RNA-Seq data that supports the findings of this study was deposited in the BioProjects database with ID number PRJNA1397219.

## Author contributions

MT planned and performed most of the experiments described here. ST and YP carried out the EEG analyses. PLF assisted with the histological analysis of the mice, while PC and JNB acquired the brain tumor tissue for this study. AYK was involved in animal husbandry and genotyping mice for the study, YRH assisted with RNA stability assays and KA analyzed the RNA-Seq data bioinformatically. DCD provided intellectual input and helped prepare the manuscript. URM conceptualized the experiments, directed the project, analyzed, and interpreted the data. URM and MT wrote the manuscript with input from all authors.

## Conflict of interest

MT and URM are inventors on a provisional patent application by Columbia University describing the use of the technology reported in this study. KA is an employee of Sarepta Therapeutics Inc.

## Funding support

This work was funded in part by the NIH and is subject to the NIH Public Access Policy. Our acceptance of federal funding gives NIH the right to make the study publicly available in PubMed Central.

- GLUT1 Deficiency, Crofoot Family and the Hope for Children Research Foundations (URM, DCD).
- AFM-France (AFM-28864), Sarepta Therapeutics Inc. and the Orphan Disease Center, University of Pennsylvania (URM).
- NIH R03 NS128211 (URM), NIH R01 NS086736, R01 NS117745 and R01 NS124854 (PLF).
- NIH P30CA013696 and the Columbia University Cancer Center (PC, JNB).
- Columbia Precision Medicine Initiative (YP).

## Supplementary Material

Supplemental data

Unedited blot and gel images

Supplemental tables 1-3

Supporting data values

## Figures and Tables

**Table 1:**
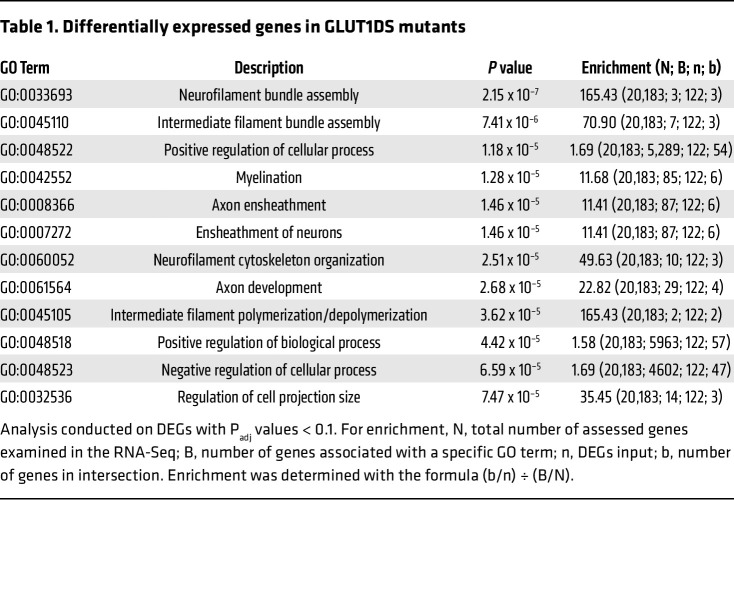
Differentially expressed genes in GLUT1DS mutants

## Version 1 03/05/2026

In-Press Preview

## Version 2 05/01/2026

Electronic publication
