# Supplementary material for: A therapeutic role for a regulatory GLUT1–associated lncRNA in GLUT1-deficient mice
Source: J Clin Invest. 2026 Mar 5;136(9):e193519. doi: 10.1172/JCI193519 (PMC13132390; doi:10.1172/JCI193519)
Supplement: Supplemental data [file jci-136-193519-s324.pdf]

A Therapeutic Role for a Regulatory Glucose Transporter1 (GLUT1)-associated lncRNA in GLUT1-deficient Mice

Maoxue Tang[1,2,3*], Sasa Teng[1,4], Yueqing Peng[1,4], Ashley Y. Kim[1,2,3], Yoon-Ra Her[1,2,3], Peter Canoll[4,5], Jeffrey N. Bruce[5], Phyllis L. Faust[4], Kailash Adhikari[6], Darryl C. De Vivo[1,2,3] and Umrao R. Monani[1,2,3,4*]

Department of [1]Neurology, [2]Center for Motor Neuron Biology and Disease and [3]Colleen Giblin Research Laboratories, Columbia University Irving Medical Center, New York, NY 10032, [4]Department of Pathology & Cell Biology, Columbia University Irving Medical Center, New York, NY 10032, [5]Department of Neurological Surgery, Columbia University Irving Medical Center, New York, NY 10032, [6]Sarepta Therapeutics, Inc. Cambridge, MA 02142

Conflict of interest: M.T. and U.R.M. are inventors on a provisional patent application by Columbia University describing the use of the technology reported in this study. K.A. is an employee of Sarepta Therapeutics Inc.

*Corresponding authors: Maoxue Tang, Umrao R. Monani

Mailing address: P&S, Room 5-422, 630 W. 168th St., New York, NY 10032
Phone: 212-342-5132
Fax: 212-342-4512
Email: Umrao R. Monani um2105@columbia.edu Maoxue Tang mt2877@cumc.columbia.edu

Supplemental figure legends

Supplemental Figure 1 – The *SLC2A1-DT* lncRNA influences GLUT1 expression. (A) lncRNA expression in human fibroblasts is reduced by siRNAs. *, $P < 0.05$, Kruskal-Wallis test, n = 3 experiments for each condition. **(B)** Representative western blot of GLUT1 protein in fibroblasts treated either with vehicle or the scrambled siRNA to *SLC2A1-DT*. **(C)** Quantification of GLUT1 in blot depicted in panel B. N.S. – not significant, *t* test, n = 4 replicates. **(D)** Representative western blot of GLUT1 protein in fibroblasts treated with either vehicle or the empty plasmid (pcDNA3.1) used to express the lncRNA. **(E)** Quantification of GLUT1 in blot depicted in panel C. N.S. – not significant, *t* test, n = 4 replicates. **(F)** Quantified results of *SLC2A1-DT* knockdown by siRNA #2 in human brain endothelial cells (BECs). **, $P < 0.01$, *t* test, n ≥ 3 replicates. **(G)** Knockdown of *SLC2A1-DT* suppressed *SLC2A1* expression significantly in human BECs. ***, $P < 0.001$, *t* test, n = 4 replicates. **(H)** Over-expressing *SLC2A1-DT* raised *SLC2A1* RNA levels in human BECs. **, $P < 0.01$, *t* test, n = 4 replicates. **(I)** A human *SLC2A1* promoter but not promoterless construct drives robust reporter expression. ***, $P < 0.001$, *t* test, n = 3 replicates. **(J)** Graph depicting increased reporter activity in HEK293 cells co-transfected with a construct expressing *SLC2A1-DT*. **, $P < 0.001$, *t* test, n ≥ 3 replicates each. Note: Luciferase in the reporter construct is driven by a 6kb *SLC2A1* promoter. An increase in **(K)** GLUT1 expression in human brain tumor samples is mirrored by a similar increase **(L)** in lncRNA levels. **, $P < 0.01$, one-way ANOVA, n ≥ 3 samples in each instance.

Supplemental Figure 2 – Representative photomicrographs of fibroblasts illustrating the localization of the *SLC2A1-DT* lncRNA. Arrows depict lncRNA puncta in the cell soma whereas the arrowhead highlights the transcript in the nucleus.

Supplemental Figure 3 – Transgenic expression and disease amelioration by the *SLC2A1-DT* lncRNA. (A) Cartoon depicting the structure of the transgene bearing the *SLC2A1-DT* lncRNA. Note: Truncation of the human *SLC2A1* gene in the transgene renders *SLC2A1* non-functional. **(B)** Representative gel following qualitative PCR depicts expression of the lncRNA transcript in transgenic mice but not in littermates devoid of the transgene. Bands correspond to gene segments amplified using primers lying in exons 1 and 2 or in exons 2 and 3. **(C)** Western blot illustrating an increase in murine GLUT1 protein in transgenic mice expressing the *SLC2A1-DT* lncRNA

transgene. **(D)** Graph depicting normalization of CSF lactate levels in GLUT1DS mutant mice expressing the *SLC2A1-DT* lncRNA transgene. ***, $P < 0.001$, one-way ANOVA, n ≥ 4 mice of each cohort.

Supplemental Figure 4 – The AAV-*SLC2A1-DT* lncRNA mitigates the neuroinflammatory response in GLUT1DS model mice. (A) Representative thalamic sections, imaged at low magnification, from 4 – 5-month-old controls and mutants treated either with AAV-eGFP or AAV-lncRNA and stained with antibodies against Iba1 and GFAP to assess gliosis in the various cohorts of mice. Note profound neuroinflammation in mutants treated with the AAV-eGFP construct; cerebral gliosis is reduced in GLUT1DS mice administered the lncRNA-containing AAV vector.

Supplemental Figure 5 – The AAV-*SLC2A1-DT* lncRNA mitigates cerebral pathology in GLUT1DS model mice. (A) Representative thalamic sections, imaged at low magnification, from 4 – 5-month-old control and mutants treated either with AAV-eGFP or AAV-lncRNA and stained with fluorescein-linked lectin to reveal the brain microvasculature. Note fewer brain capillaries in the AAV-eGFP-treated individual and relative normalization of brain angiogenesis in the mutants treated with the lncRNA; intense fluorescence in mutants injected with AAV-eGFP derives from reporter expressed in cells of the neuropil **(B)** Representative EEG spectrogram (at 0-25Hz) and corresponding trace below it depicting a convulsive seizure in a GLUT1DS mutant delivered the control (AAV-eGFP) vector. These were eliminated in mutants treated with the AAV-lncRNA construct.

Supplemental Figure 6 – Early administration of the AAV-*SLC2A1-DT* lncRNA does not trigger major organ pathology in model mice. Hematoxylin/eosin histochemistry of the major organs of the body at 12 months of age depicts normal cellular morphology in most tissues of mutants treated with AAV-lncRNA. Cholestatic bile pigment in liver of lncRNA treated mice (arrowhead) was also observed in AAV-eGFP-treated mutants

Supplemental Information

Rotarod test. The rotarod test was administered by subjecting mice to a training period of 5mins. on an accelerating rotarod from 0-40rpm (Ugo Basile Inc., Italy) three times a day for four consecutive days. Performance on day 5 was assessed at a setting of 25rpm. The rotarod was cleaned and dried prior to testing. Latency to fall off the rotating rod was recorded and the experiment terminated if a mouse surpassed 1000s.

Preparation of brain parenchymal and vessel fractions. Following euthanasia and perfusion with 1X PBS, mouse brains were extracted and gently homogenized using a Dounce-type glass homogenizer in 1ml PBS. The resulting extract was centrifuged at 1000g for 5min., the pellet re-suspended once again in 1ml 1X PBS and the centrifugation step repeated. The supernatant was then discarded and the pellet re-suspended (1ml 18% dextran solution in PBS). This suspension was centrifuged (10,000g for 1min.), the pellet retrieved, and the supernatant containing the neuropil fraction transferred to new tube. The pellet was once again re-suspended (1ml 18% dextran in PBS) and the centrifugation repeated. This process was repeated a third time and the pellets containing the vessel fractions were pooled and stored at -80°C until use. The supernatant fractions – containing the neuropil – were similarly combined and stored for analysis.

Vectorette PCR. To map the insertion site of the transgene bearing the human *SLC2A1* locus, we used a vectorette PCR protocol previously described in references 50 and 51. In brief, genomic DNA from transgenic mice was digested individually with each of the following enzymes – RsaI, HincII, HpaI and ScaI – to create corresponding libraries. Subsequently, a linker (vectorette) consisting of 52bp strands that exhibit complementarity at their ends but not in a central 29bp central region was ligated onto blunt ends generated by the various restriction enzymes. Such libraries were then subjected to an initial round of PCR using a transgene specific primer and a vectorette primer (UVP) that binds to sequence in the “bubble” formed within the vectorette. Amplification products were then used as template for a nested PCR using the vectorette primer and a second transgene specific primer. This increases specificity of the PCR and generally resulted in one distinct product. In instances where the nested PCR did not result in a single distinct PCR product, a double-nested PCR was carried out using amplification products from the nested PCR as template. The final PCR products were then sequenced, junction fragments determined

and sequence in these fragments corresponding to the mouse genome mapped onto the murine reference genome (GRCM39) to identify the precise insertion site of the transgene.

Quantitative PCR. RNA for quantitative RT-PCR was extracted from relevant tissue or cells using Trizol (Life Technologies, Carlsbad, CA) according to the manufacturer's instructions. Following treatment with DNAse, cDNA was synthesized using the RevertAid First Strand cDNA Synthesis Kit (Thermo Scientific). QPCRs were carried out on a CFX96 Real-Time Systems PCR analyzer (Bio-Rad Labs) using the SsoAdvanced Universal SYBR Green Supermix (Bio-Rad Labs). Relative expression of the *SLC2A1* gene and the *SLC2A1-DT* lncRNA was calculated using the ΔΔCT method. Absolute amounts of *SLC2A1* and *SLC2A1-DT* RNAs in brain tumor samples were measured following construction of a standard curve. These standard curves were constructed using serial 10-fold dilutions of purified plasmid containing the target sequence. Linear regression was used to calculate the slope and intercept for the standard curve.

Key Reagents

PCR primers

Primer	SOURCE	SEQUENCE
Primers for PCR		
SLC2A1-DT Exon1-3 L	IDT	5'-CTCTTGCCTGGAAATGCATAC-3'
SLC2A1-DT Exon1-3 R	IDT	5'-CTTCTCTGGGCCTCAGTTTG-3'
SLC2A1-DT Exon2-3 L	IDT	5'-TCCTGGCCCTGACTTTTCTA-3'
SLC2A1-DT Exon2-3 R	IDT	5'-GTCCAGCCCTGCAGATATTC-3'
mSMN F	IDT	5'-GGTGCTAGGATCTCTGTGTTCGTGC-3'
mSMN R	IDT	5'-GGTCCCACCACCTAAGAAAGC-3'
Primers for qRT-PCR		
Mouse Slc2a1 Fwd	IDT	5'-CTTGCTTGTAGAGTGACGATC-3'
Mouse Slc2a1 Rvs	IDT	5'-CAGTGATCCGAGCACTGCTC-3'
Mouse GAPDH Fwd	IDT	5'- AATGTGTCCGTCGTGGATCTGA-3'
Mouse GAPDH Rvs	IDT	5'- GATGCCTGCTTCACCACCTTCT-3'
β-actin Fwd	IDT	5'-TGTTACCAACTGGGACGACA-3'
β-actin Rvs	IDT	5'-GGGGTGTTGAAGGTCTCAAA-3'
SLC2A1-DT Exon1-2 L2	IDT	5'-GGGATTGACTTCTCCCTTCC-3'
SLC2A1-DT Exon1-2 R2	IDT	5'- TGTTCCTACCAGCTCCTCTCA-3'
Human SLC2A1 EX1-2-L1	IDT	5'- GTCGGAGTCAGAGTCGCAGT-3'

Human SLC2A1 EX1-2-R1	IDT	5’- GGCATTGATGACTCCAGTGTT-3’
Primers for vectorette PCR		
Vectorette upper strand	IDT	5’-AAGGAGAGGACGCTGTCTGTCGAAGGTAAGGAACGGACGAGAG AAGGGAGAG-3’
Vectorette lower strand	IDT	5’-CTCTCCCTTCTCGAATCGTAACCGTTCGTACGAGAATCGCTGTCC TCTCCTT-3’
Universal vectorette primer (UVP)	IDT	5'CGAATCGTAACCGTTCGTACGAGAATCGCT-3'
2829 Rev (Transgene specific primer)	IDT	5’- CCCACTAGCCAGTAGTTGGCC-3’
ClaI nested (Transgene specific primer)	IDT	5’- GGAGCAGGTGACAGCTATGAC-3’
ClaI dbl-nested (Transgene specific primer)	IDT	5’- GGCTAGAAGATGAGAGGTGCAG-3’

Other reagents

REAGENT or RESOURCE	SOURCE	IDENTIFIER
siRNAs for knockdown experiments		
SLC2A1-DT DsiRNA #1 (5'-3') (Duplex Sequences)	IDT	rGrUrArArArGrGrArUrUrGrArUrGrArGrArArArA rGrCrAGA
		rUrCrUrGrCrUrUrUrUrCrUrCrArUrCrArArUrCr CrUrUrUrArCrArA
SLC2A1-DT DsiRNA #2 (5'-3') (Duplex Sequences)	IDT	rCrArCrUrUrCrArCrArGrArUrGrArArGrCrArArA rCrUrGAG
		rCrUrCrArGrUrUrUrGrCrUrUrCrArUrCrUrGrUr GrArArGrUrGrGrG
SLC2A1-DT DsiRNA #6 (5'-3') (Duplex Sequences)	IDT	rGrArCrUrUrUrUrCrUrArArGrArUrGrArGrArGr GrArGrCTG
		rCrArGrCrUrCrCrUrCrUrCrArUrCrUrUrArGrAr ArArArGrUrCrArG
Scrambled DsiRNA (5'-3') (Duplex Sequences)	IDT	rUrArArCrGrGrUrArArCrGrUrGrArUrCrCrUrGr ArArUrGGC
		rArCrGrCrCrArUrUrCrArGrGrArUrCrArCrGrUr UrArCrCrGrUrUrA
Antibodies for Western blotting		
GLUT1 (1:5000)	Millipore	Cat#07-1401
Vinculin (1:5000)	Abcam	Cat#ab129002
Goat anti-rabbit IgG (1:10,000)	Invitrogen	Cat#31460
Goat anti-mouse IgG (1:10,000)	Jackson Immunoresearch	Cat#115-035-003
SMN (1:5000)	BD Biosciences	Cat# 610640
Antibodies for Immunostaining		
Lectin (1:1000)	Vector Laboratories	Cat#FL-1171
GFAP (1:500)	Abcam	Cat#ab134436
Iba-1 (1:500)	Wako	Cat#019-19741
Alexa Fluor 488 goat α-rabbit (1:1000)	Abcam	Cat#ab150085

Alexa Fluor 647 goat α-chicken (1:1000)	Abcam	Cat#ab150171
Experimental Models: Organisms/Strains		
Slc2a1$^{+/-}$ mouse model	Giblin Labs, Columbia University	N/A
SLC2A1-DT^{Tg} transgenic mice	Giblin Labs, Columbia University	N/A
Human Tissues	New York Brain Bank	N/A
Glioblastoma samples	Bartoli Laboratory Tumor Bank, Columbia University Medical Center	N/A
Oligodendroglioma samples	Bartoli Laboratory Tumor Bank, Columbia University Medical Center	N/A
Human Primary Brain Microvascular Endothelial Cells	Cell Biologics	Cat# H-6023
Human fibroblasts	Giblin Labs, Columbia University	N/A
scAAV-PHP.eb-SLC2A1-DT virus	Horae Gene Therapy vector core, U.Mass.	N/A
scAAV-PHP.eb-GFP virus	Horae Gene Therapy vector core, U.Mass.	N/A
Software and Algorithms		
GraphPad Prism	Graph Pad Software	https://www.graphpad.com/scientificsoftware/prism/
ImageJ	NIH	https://imagej.nih.gov/ij/
Leica LAS X	Leica	https://www.leica-microsystems.com/

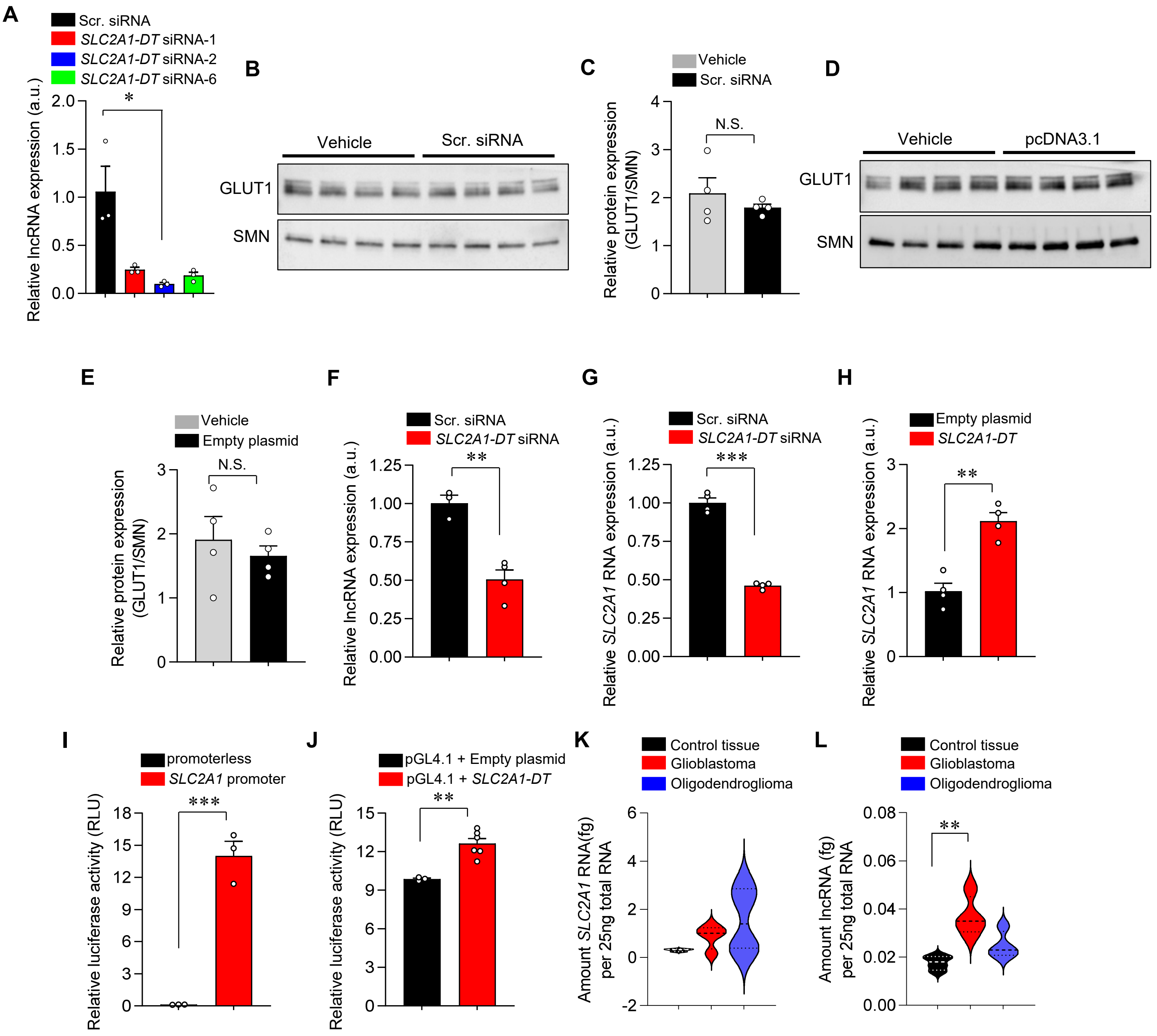

Supplemental Figure 1 – The *SLC2A1-DT* lncRNA influences *SLC2A1* expression. (A) lncRNA expression in human fibroblasts is reduced by siRNAs. *, $P < 0.05$, Kruskal-Wallis test, n = 3 experiments for each condition. **(B)** Representative western blot of GLUT1 protein in fibroblasts treated either with vehicle or the scrambled siRNA to *SLC2A1-DT*. **(C)** Quantification of GLUT1 in blot depicted in panel B. N.S. – not significant, *t* test, n = 4 replicates. **(D)** Representative western blot of GLUT1 protein in fibroblasts treated with either vehicle or the empty plasmid (pcDNA3.1) used to express the lncRNA. **(E)** Quantification of GLUT1 in blot depicted in panel C. N.S. – not significant, *t* test, n = 4 replicates **(F)** Quantified results of *SLC2A1-DT* knockdown by siRNA #2 in human brain endothelial cells (BECs). **, $P < 0.01$, *t* test, n ≥ 3 replicates. **(G)** Knockdown of *SLC2A1-DT* suppressed *SLC2A1* expression significantly in human BECs. ***, $P < 0.001$, *t* test, n = 4 replicates. **(H)** Over-expressing *SLC2A1-DT* raised *SLC2A1* RNA levels in human BECs. **, $P < 0.01$, *t* test, n = 4 replicates. **(I)** A human *SLC2A1* promoter but not promoterless construct drives robust reporter expression. ***, $P < 0.001$, *t* test, n = 3 replicates. **(J)** Graph depicting increased reporter activity in HEK293 cells co-transfected with a construct expressing *SLC2A1-DT*. **, $P < 0.001$, *t* test, n ≥ 3 replicates each. Note: Luciferase in the reporter construct is driven by a 6kb *SLC2A1* promoter. An increase in **(K)** *SLC2A1* expression in human brain tumor samples is mirrored by a similar increase **(L)** in lncRNA levels. **, $P < 0.01$, one-way ANOVA, n ≥ 3 samples in each instance.

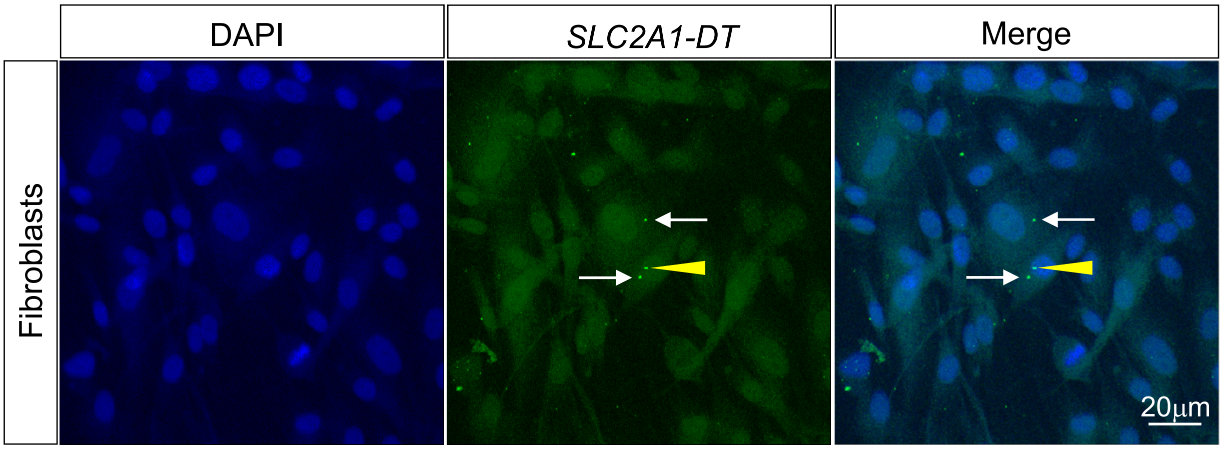

Supplemental Figure 2 – Representative photomicrographs of fibroblasts illustrating the localization of the *SLC2A1-DT* lncRNA. Arrows depict lncRNA puncta in the cell soma whereas the arrowhead highlights the transcript in the nucleus.

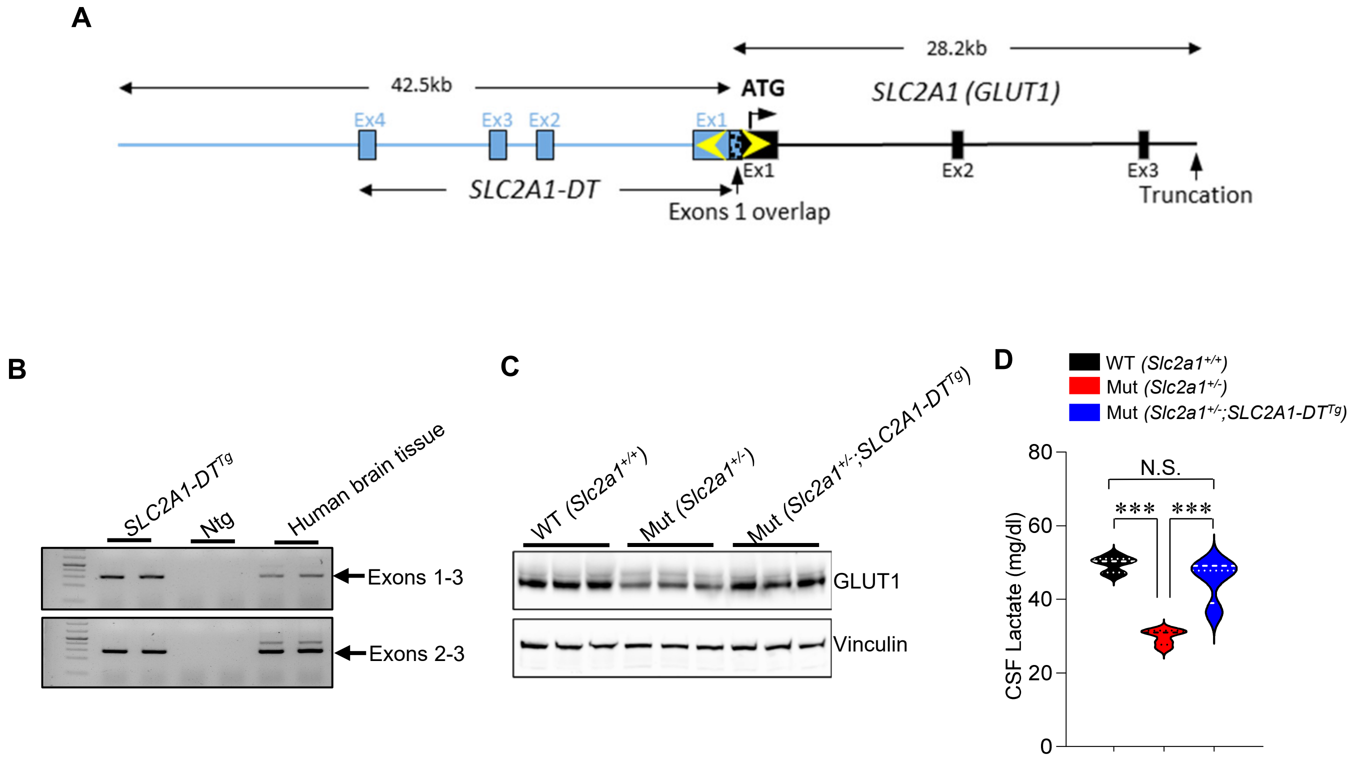

Supplemental Figure 3 – Transgenic expression and disease amelioration by the *SLC2A1-DT* lncRNA. **(A)** Cartoon depicting the structure of the transgene bearing the *SLC2A1-DT* lncRNA. Note: Truncation of the human *SLC2A1* gene in the transgene renders *SLC2A1* non-functional. **(B)** Representative gel following qualitative PCR depicts expression of the lncRNA transcript in transgenic mice but not in littermates devoid of the transgene. Bands correspond to gene segments amplified using primers lying in exons 1 and 2 or in exons 2 and 3. **(C)** Western blot illustrating an increase in murine GLUT1 protein in transgenic mice expressing the *SLC2A1-DT* lncRNA transgene. **(D)** Graph depicting normalization of CSF lactate levels in GLUT1DS mutant mice expressing the *SLC2A1-DT* lncRNA transgene. ***, $P < 0.001$, one-way ANOVA, n ≥ 4 mice of each cohort.

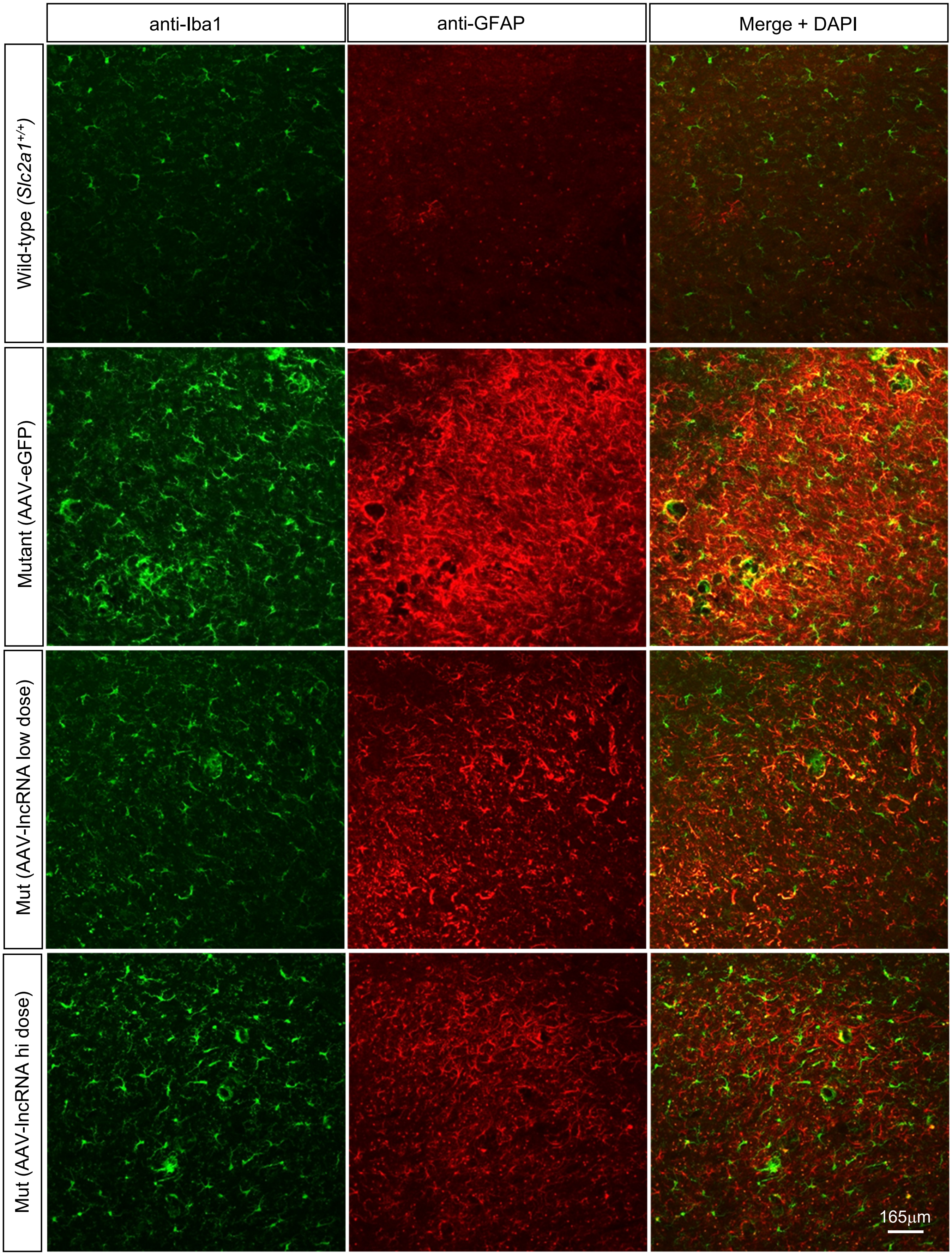

Supplemental Figure 4 – The AAV-*SLC2A1-DT* lncRNA mitigates the neuroinflammatory response in GLUT1DS model mice. (A) Representative thalamic sections, imaged at low magnification, from 4 – 5-month-old controls and mutants treated either with AAV-eGFP or AAV-lncRNA and stained with antibodies against Iba1 and GFAP to assess gliosis in the various cohorts of mice. Note profound neuroinflammation in mutants treated with the AAV-eGFP construct; cerebral gliosis is reduced in Glut1DS mice administered the lncRNA-containing AAV vector.

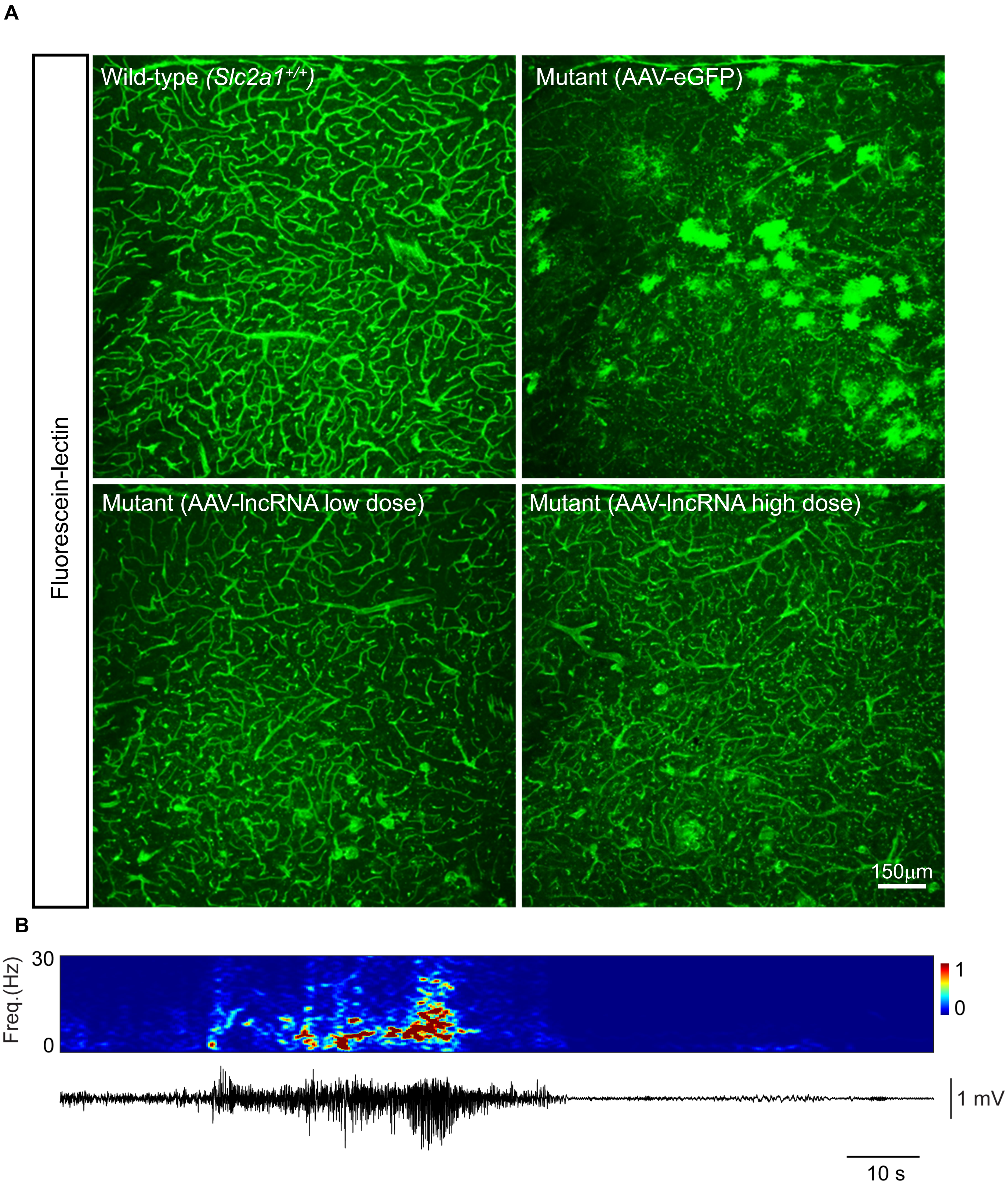

Supplemental Figure 5 – The AAV-*SLC2A1-DT* lncRNA mitigates cerebral pathology in GLUT1DS model mice. (A) Representative thalamic sections, imaged at low magnification, from 4 – 5-month-old control and mutants treated either with AAV-eGFP or AAV-lncRNA and stained with fluorescein-linked lectin to reveal the brain microvasculature. Note fewer brain capillaries in the AAV-eGFP-treated individual and relative normalization of brain angiogenesis in the mutants treated with the lncRNA; intense fluorescence in mutants injected with AAV-eGFP derives from reporter expressed in cells of the neuropil **(B)** Representative EEG spectrogram (at 0-30Hz) and corresponding trace of 2min. duration below it depicting a convulsive seizure in a GLUT1DS mutant delivered the control (AAV-eGFP) vector. These were eliminated in mutants treated with the AAV-lncRNA construct.

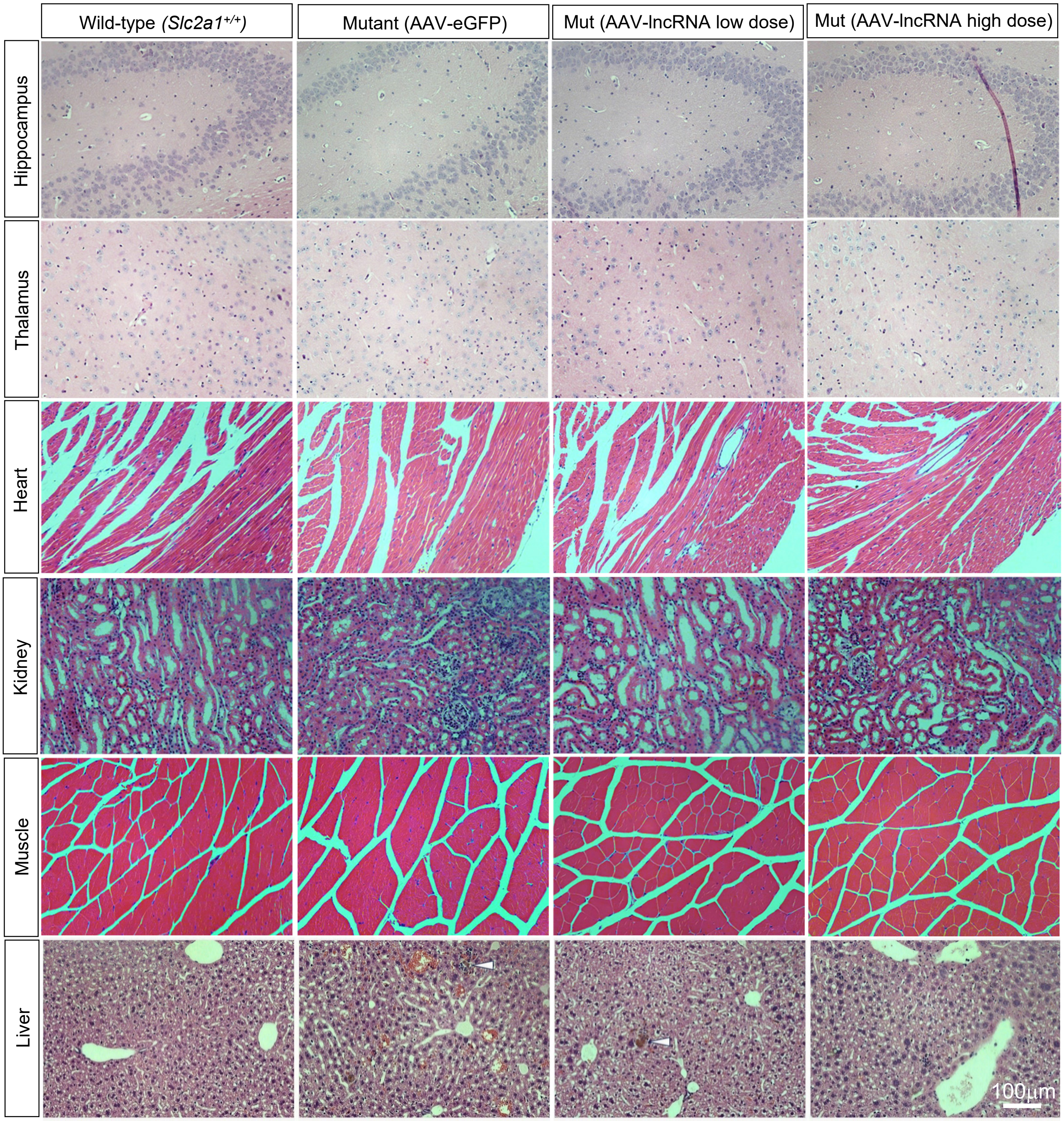

Supplemental Figure 6 – Early administration of the AAV-*SLC2A1-DT* lncRNA does not trigger major organ pathology in model mice. Hematoxylin/eosin histochemistry of the major organs of the body at 12 months of age depicts normal cellular morphology in most tissues of mutants treated with AAV-lncRNA. Cholestatic bile pigment in liver of lncRNA treated mice (arrowhead) was also observed in AAV-eGFP-treated mutants.

Supplemental Table 4 – Prevalence and characteristics of natural antisense transcripts in the *SLC2A1 (GLUT1)* locus of diverse species.

Organism *(species)*	Presence/Absence of NAT	NAT genomic size (kb)	Processed NAT size (bp)	NAT spliced (or not)	Number of NAT exons
Humans *(Homo sapiens)*	✓	24.3	1124	✓	4
Chimp *(Pan troglodytes)*	✓	15.0	770	✓	3
Baboon *(Papio Anubis)*	✓	14.5	1537	✓	4
Macaque *(Macaca mulatta)*	✓	35.5	1444	✓	4
Pig *(Sus scrofa)*	✓	13.8	2755	✓	2
Cow *(Bos taurus)*	✓	9.7	8640	✓	2
Dog *(Canis lupus)*	✓	3.7	3677	✗	1
Cat *(Felis catus)*	✓	16.5	2553	✓	3
Rat *(Rattus norvegicus)*	✓	9.9	2692	✓	4
Mouse *(Mus musculus)*	✓	8.9	1754	✓	2
Dolphin *(Tursiops truncatus)*	✓	2.9	1704	✓	3
Chicken *(Gallus gallus)*	✗	-	-	-	-

Note: Search conducted on https://www.ncbi.nlm.nih.gov/ on 1-19-2025.
