# Supplementary material for: A therapeutic role for a regulatory GLUT1–associated lncRNA in GLUT1-deficient mice
Source: J Clin Invest. 2026 Mar 5;136(9):e193519. doi: 10.1172/JCI193519 (PMC13132390; doi:10.1172/JCI193519)
Supplement: Unedited blot and gel images [file jci-136-193519-s325.pdf]

Unedited Gels for Figure 1C

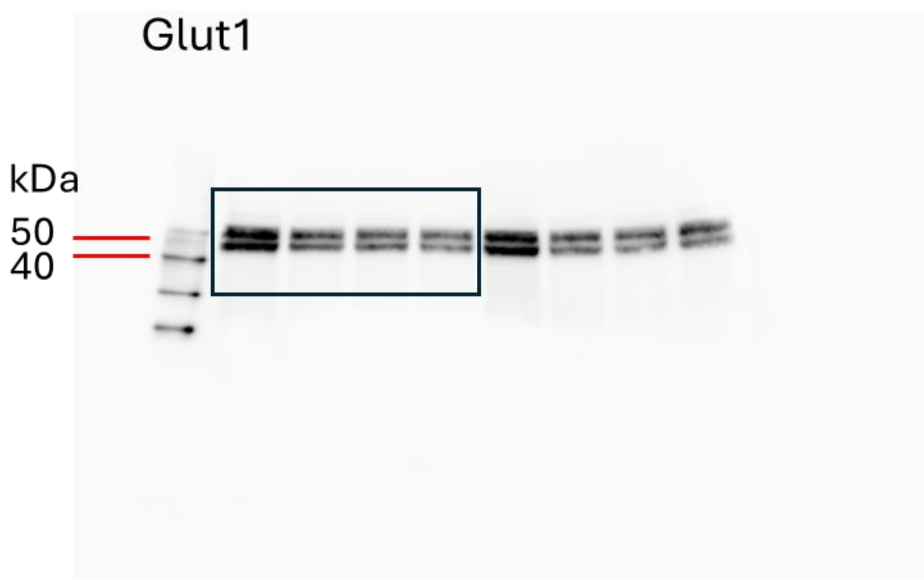

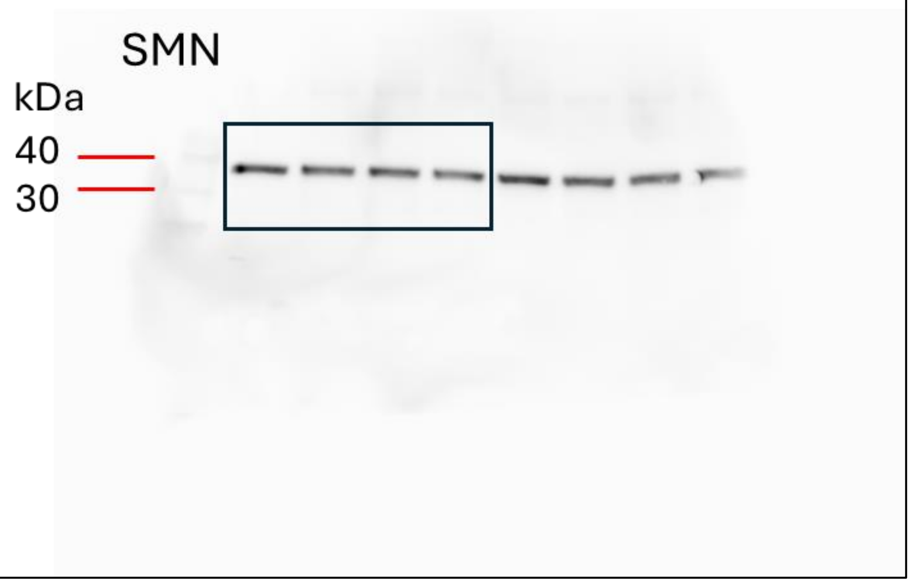

Unedited Gels for Figure 1E

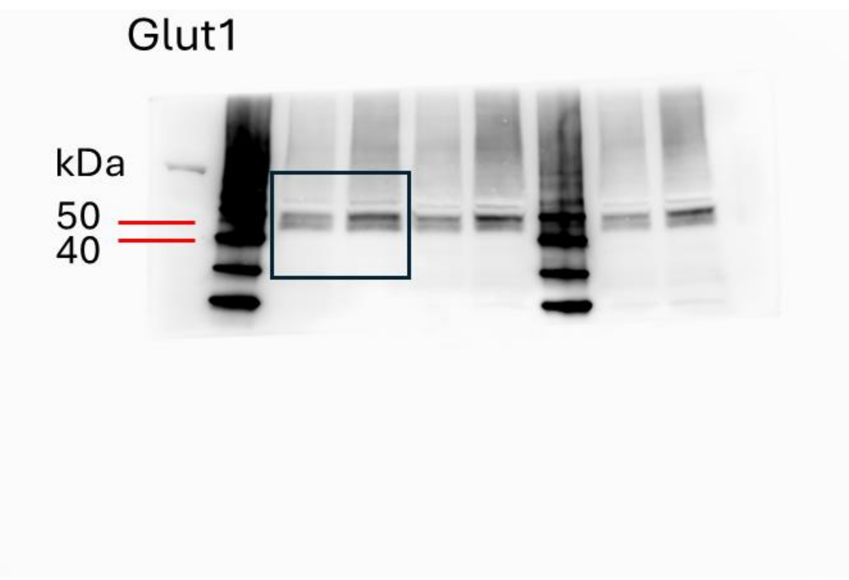

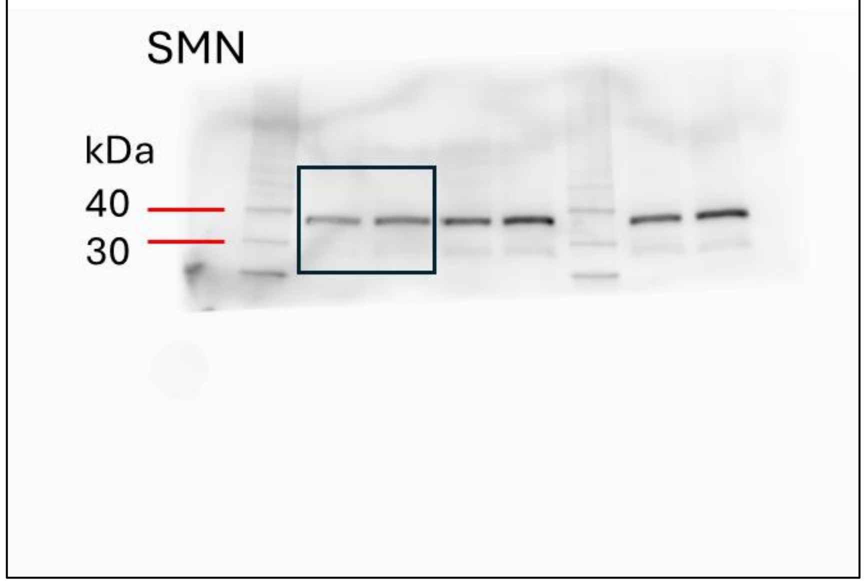

Unedited Gels for Figure 4C

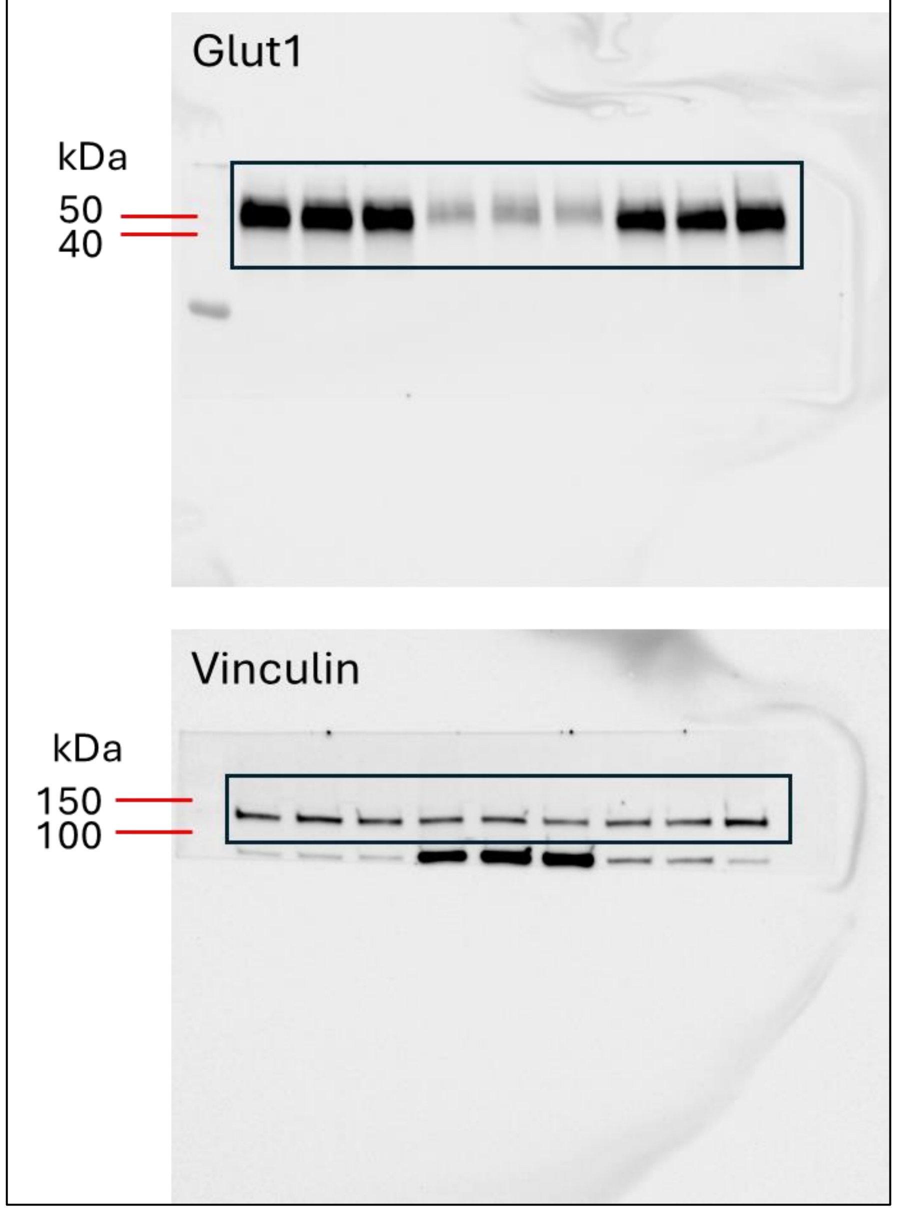

Unedited Gels for Figure 4E

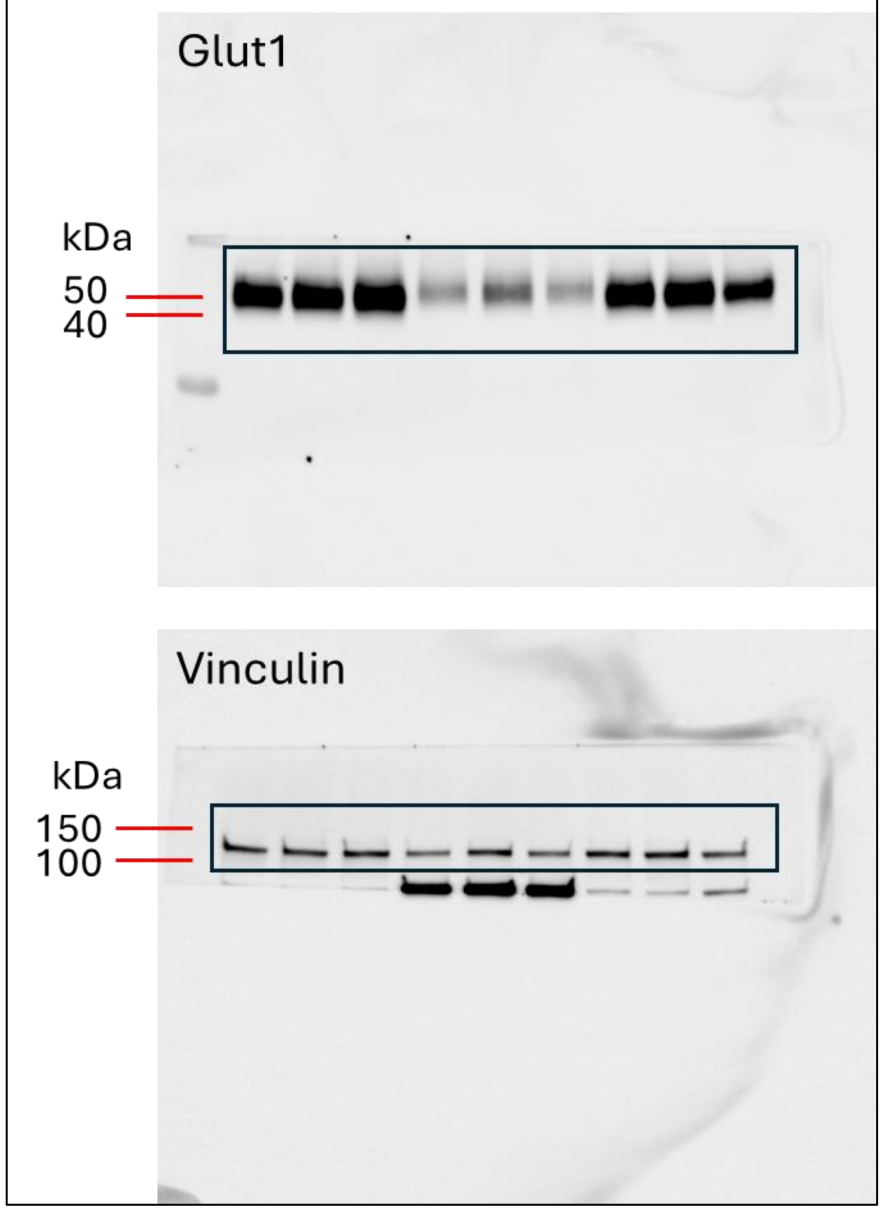

Unedited Gels for Supplemental Figure. 1B

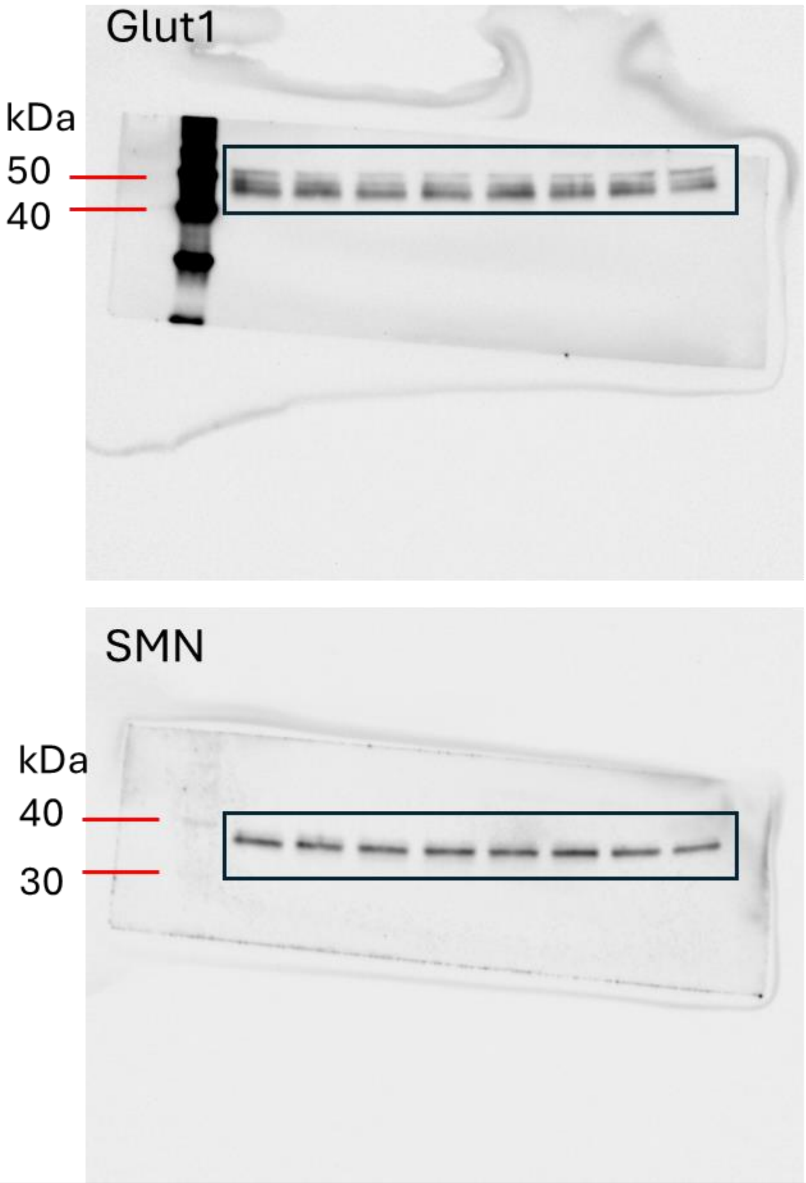

Unedited Gels for Supplemental Figure. 1D

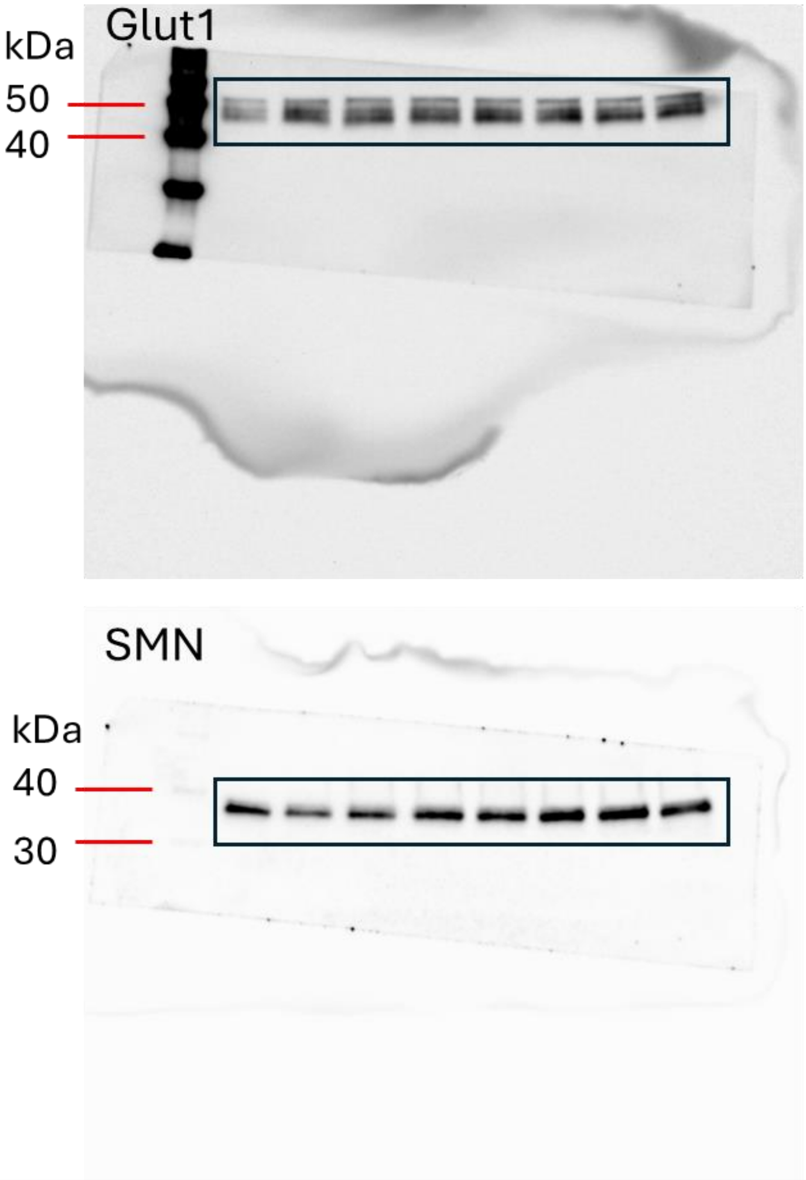

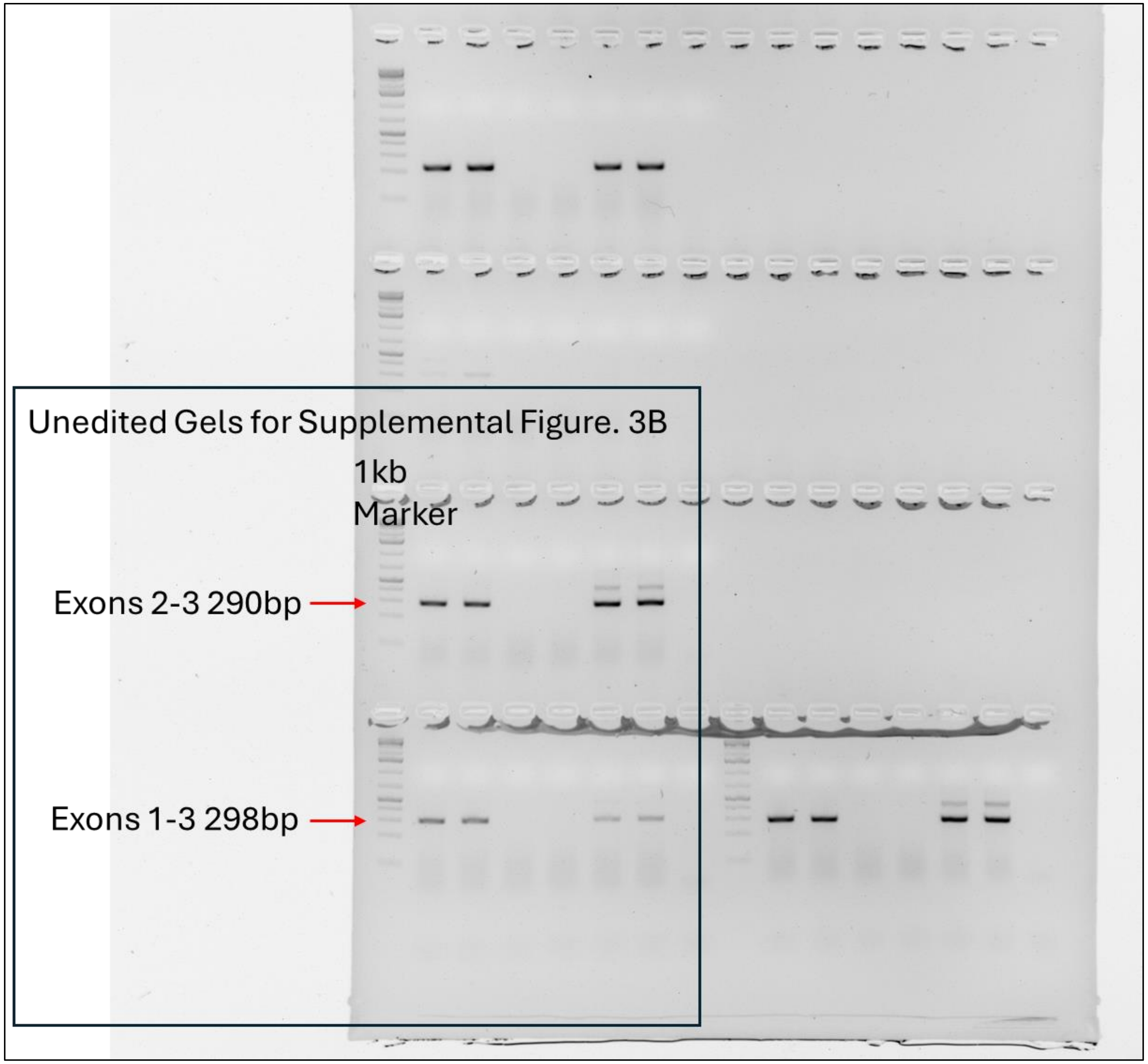
Unedited Gels for Supplemental Figure. 3B
1kb
Marker
Exons 2-3 290bp
Exons 1-3 298bp

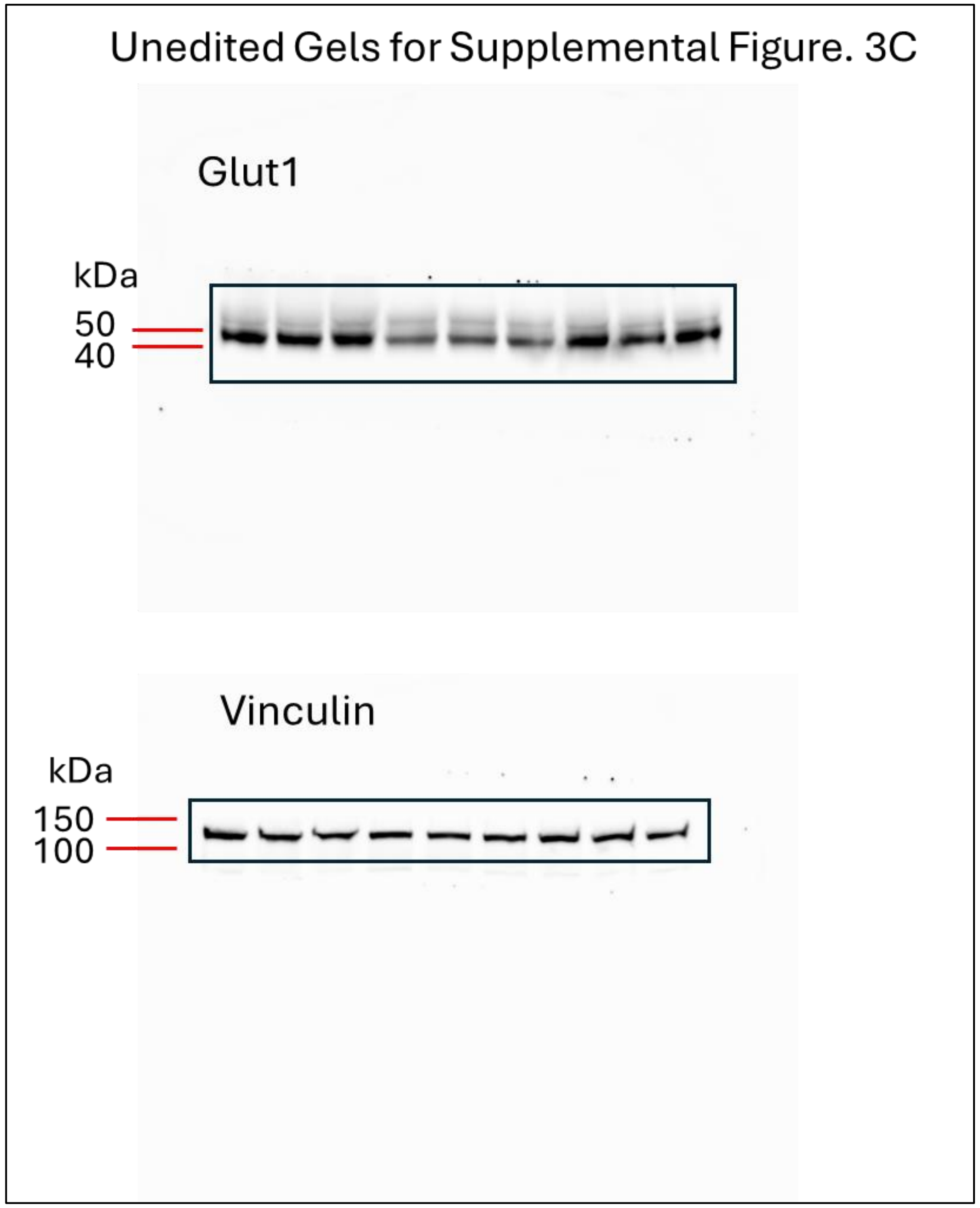
Unedited Gels for Supplemental Figure. 3C
Glut1
kDa
50
40
Vinculin
kDa
150
100
